# Combining *ERAP1* silencing and entinostat therapy to overcome resistance to cancer immunotherapy in neuroblastoma

**DOI:** 10.1186/s13046-024-03180-y

**Published:** 2024-10-22

**Authors:** Patrizia Tempora, Silvia D’Amico, Paula Gragera, Verena Damiani, Kamila Krol, Valentina Scaldaferri, Kirti Pandey, Shanzou Chung, Valeria Lucarini, Ezio Giorda, Marco Scarsella, Gabriele Volpe, Marco Pezzullo, Cristiano De Stefanis, Valentina D’Oria, Lorenzo De Angelis, Roberto Giovannoni, Maria Antonietta De Ioris, Ombretta Melaiu, Anthony W. Purcell, Franco Locatelli, Doriana Fruci

**Affiliations:** 1 Bambino Gesù Children’s Hospital, IRCCS, Rome, Italy; 2 Department of Biochemistry and Molecular Biology, Infection and Immunity Program, Biomedicine Discovery Institute, Monash University, Clayton 3800, Victoria, Australia; 3 Plaisant Polo Tecnologico S.R.L, Castel Romano, Rome, Italy; 4 Department of Biology, Genetic Unit, University of Pisa, Pisa, Italy; 5 Department of Clinical Sciences and Translational Medicine, University of Rome “Tor Vergata”, Rome, Italy; 6 Department of Life Sciences, Catholic University of the Sacred Heart, Rome, Italy

**Keywords:** Neuroblastoma, ERAP1, Antigen processing, Cancer immunotherapy, Epigenetic, Combination therapy, Immunopeptidome

## Abstract

**Background:**

Checkpoint immunotherapy unleashes tumor control by T cells, but it is undermined in non-immunogenic tumors, e.g. with low MHC class I expression and low neoantigen burden, such as neuroblastoma (NB). Endoplasmic reticulum aminopeptidase 1 (ERAP1) is an enzyme that trims peptides before loading on MHC class I molecules. Inhibition of ERAP1 results in the generation of new antigens able of inducing potent anti-tumor immune responses. Here, we identify a novel non-toxic combinatorial strategy based on genetic inhibition of ERAP1 and administration of the HDAC inhibitor (HDACi) entinostat that increase the immunogenicity of NB, making it responsive to PD-1 therapy.

**Methods:**

CRISPR/Cas9-mediated gene editing was used to knockout (KO) the *ERAP1* gene in 9464D NB cells derived from spontaneous tumors of TH-MYCN transgenic mice. The expression of MHC class I and PD-L1 was evaluated by flow cytometry (FC). The immunopeptidome of these cells was studied by mass spectrometry. Cocultures of splenocytes derived from 9464D bearing mice and tumor cells allowed the assessment of the effect of ERAP1 inhibition on the secretion of inflammatory cytokines and activation and migration of immune cells towards ERAP1 KO cells by FC. Tumor cell killing was evaluated by Caspase 3/7 assay and flow cytometry analysis. The effect of ERAP1 inhibition on the immune content of tumors was analyzed by FC, immunohistochemistry and multiple immunofluorescence.

**Results:**

We found that inhibition of ERAP1 makes 9464D cells more susceptible to immune cell-mediated killing by increasing both the recall and activation of CD4^+^ and CD8^+^ T cells and NK cells. Treatment with entinostat induces the expression of MHC class I and PD-L1 molecules in 9464D both in vitro and in vivo. This results in pronounced changes in the immunopeptidome induced by ERAP1 inhibition, but also restrains the growth of ERAP1 KO tumors in vivo by remodelling the tumor-infiltrating T-cell compartment. Interestingly, the absence of ERAP1 in combination with entinostat and PD-1 blockade overcomes resistance to PD-1 immunotherapy and increases host survival.

**Conclusions:**

These findings demonstrate that ERAP1 inhibition combined with HDACi entinostat treatment and PD-1 blockade remodels the immune landscape of a non-immunogenic tumor such as NB, making it responsive to checkpoint immunotherapy.

**Supplementary Information:**

The online version contains supplementary material available at 10.1186/s13046-024-03180-y.

## Background

Immunotherapeutic approaches, such as immune checkpoint inhibition (ICI), have been shown to be remarkably effective in a variety of adult cancers, but non-resolving in pediatric cancers [1, 2].

Neuroblastoma (NB) is the most common extracranial pediatric solid tumor and the leading cause of death in childhood cancers [3, 4]. NB arises from primitive neural crest cells of the developing sympathetic nervous system, and is fatal in half of all patients with high-risk NB at diagnosis, despite intensive therapy [5–7]. Major advances in the treatment of patients with high-risk NB have been achieved with the addition to first-line maintenance therapy of dinutuximab and dinutuximab β antibodies targeting the disialoganglioside GD2 expressed on the surface of NB cells [8]. Children with refractory/relapse NB have a poor prognosis with an Event-Free Survival (EFS) < 10% [4]. More effective treatments with fewer side effects are therefore urgently needed. In this scenario, the immunotherapeutic approach seems a promising option, as demonstrated by the recent results of CAR T-cell immunotherapy against GD2, haploidentical stem cell transplantation and treatment with dinutuximab β antibody (Ab) [9, 10].

High-risk NBs are cold [11] and non-immunogenic tumors, i.e., with a low tumor mutational burden (TMB) [12, 13], low expression of surface MHC class I molecules [14, 15] and consequently low neoantigen presentation and weak anti-tumor reactivity of the few tumor-infiltrating lymphocytes (TIL) present. These characteristics make NB an ideal model to address the problems associated with the inability to trigger strong anti-tumor immune responses.

Low expression of surface major histocompatibility complex (MHC) class I molecules in NBs is a phenomenon resulting from epigenetic and/or post-transcriptional processes [15, 16], which can be reversed by treatment with inflammatory cytokines, such as IFNγ and TNFα [16]. However, the therapeutic applicability of these molecules is limited by their severe toxicity [17, 18]. Cornel and colleagues, using a pharmacological repurposing strategy, recently identified the histone deacetylase inhibitor (HDACi) entinostat as a safe inducer of MHC class I surface expression in human NB cell lines [19]. Interestingly, they found that the increase of MHC class I by entinostat was independent of the activation of the IFNγ/NFκB pathway, which is known to be repressed in high-risk NBs [15, 16]. However, high expression of MHC class I molecules is not sufficient to induce tumor-specific CD8^+^ T-cell responses in the absence of an adequate repertoire of immunogenic neoantigens. One way to successfully address this problem is to target the antigen processing and presentation (APP) pathway, which allows proteins to be sampled immediately after synthesis, thus quickly alerting immune cells to detect infections and tumorigenesis [20].

Endoplasmic reticulum aminopeptidase 1 (ERAP1) is a key component of the APP pathway and the prototype of a new class of molecules [20] able to reprogram immunogenicity by drastically altering the antigen repertoire presented by MHC class I molecules [21, 22]. Several studies have shown that inhibition of ERAP1 causes a profound change in the immunopeptidome able of inducing substantial anti-tumor immune responses by CD8^+^ T cells and natural killer (NK) cells resulting in the control of tumor growth [20, 23, 24]. We found that inhibition of ERAP1 in syngeneic mice causes rejection of murine T-cell lymphoma RMA by NK cells [23]. Similarly, in human tumor cells, ERAP1 inhibition leads to NK-cell activation by altering the interaction of peptide-MHC class I complexes with NK-cell inhibitory receptors [25, 26]. James [24] and Keller [27] found that ERAP1 inhibition leads to a potent antitumor cytotoxic response of CD8^+^ T cells in a mouse model of colon cancer and a human melanoma cell line, respectively. It is therefore possible that inhibition of ERAP1, by generating a novel immunopeptidome, may be a viable therapeutic strategy to enhance anti-tumor immune responses in non-immunogenic tumors, such as NB, in combination with a treatment that enhances the surface expression of MHC class I molecules.

In this work, using in vitro, ex vivo and in vivo approaches, we identified a novel combinatorial strategy that can improve the immunogenicity of a mouse model of NB. Specifically, we found that genetic inhibition of ERAP1 combined with entinostat administration, is able to i) induce the expression of MHC class I and PD-L1 molecules, ii) modify the immunopeptidome, iii) increase the recruitment of activated T cells and NK cells into the tumor microenvironment (TME) and, correspondingly, iv) overcome tumor resistance to PD-1 blockade.

## Methods

### Cell lines, reagents

The transgenic NB cell lines 9464D and 975A2 derived from spontaneous tumors arising in TH-MYCN transgenic mice on a C57BL/6 background [28] were kindly gifted by Dr. Crystal Mackall (Stanford University, CA). HEK293T (CRL-3216) cells were purchased from ATCC. Cells were grown in RPMI-1640 or DMEM supplemented with 10% FCS (Gibco) with 100U/ml Pen/Strep (Gibco) and 2 mM Glut (Gibco) under standard conditions (37 °C and 5% CO_2_) on tissue-culture treated plastic plates, passaged no more than four times since thawing and routinely tested for the absence of mycoplasma. Mouse IFNγ and human IL-2 were purchased from R&D system. Entinostat (MS-275) and anti-PD-1 Ab (clone RMPI-14) were purchased from Chemietek and Bio X Cell, respectively.

### Generation of ERAP1 knockout cell lines

*ERAP1*-targeting or scrambled (CTR) sgRNA oligonucleotides (Supplementary Table 1) were cloned into LentiCRISPRv2GFP (Addgene #82,416) backbone using standard molecular cloning. Lentiviral particles were generated by transfection of HEK293T cells with either *ERAP1*-targeting or CTR sgRNA vectors and CRISPR & MISSION lentiviral packaging mix (Sigma-Aldrich) according to manufacturer’s guidelines. Lentiviral supernatants were collected 72 h after transfection, 0.45 μm-filtered to remove floating cells and debris, and concentrated by high-speed centrifugation at 30,000 rpm for 2 h. Viral pellets were resuspended in complete RPMI medium with 8 µg/ml Hexadimethrine bromide (Sigma-Aldrich) and used for tumor cell transduction by spin-inoculation.

sgCTR- and sgERAP1-transduced cells were sorted according to green fluorescent protein (GFP) expression by using Fluorescent Activated Cell Sorting (FACS) and expanded in culture. After 15 days, cells that had spontaneously lost GFP expression were selected by cell sorting and tested for the lack of Cas9 expression (not shown). Successful knockout (KO) of ERAP1 was confirmed by Western blot and Sanger sequencing (Supplementary Table 2). Gene editing efficiency was evaluated in CRISP-ID (http://crispid.gbiomed.kuleuven.be/) and TIDE (http://tide.dfci.harvard.edu/) web tools. CRISP-ID enables the identification of the size and position of the Cas9 cleavage site, whereas TIDE provided insights into the total efficiency of gene editing, goodness-of-fit and statistical significance for each indel (*P* value) [29, 30]. Potential off-target sites were predicted using the Cas-OFFinder (http://www.rgenome.net/cas-offinder/) and verified by whole genome sequencing analysis.

### Western blot analysis

Whole-cell lysates were prepared in RIPA buffer (25 mM Tris–HCl (pH 8.8), 150 mM NaCl, 5 mM EDTA, 1% Triton X-100, 1% sodium deoxycholate and 0.1% sodium dodecyl sulfate). Lysates clarified by high-speed centrifugation were normalized using the bicinchoninic acid (BCA) Protein Assay Reagent Kit (Thermo Fisher Scientific). Equal amounts of cell lysate (30 μg/lane) were loaded onto 10 or 12% SDS-PAGE. After electrophoresis, the separated proteins were electroblotted onto a nitrocellulose membrane at 300 mA for 2 h using a transfer buffer (25 mM Tris, 192 mM glycine and 10% methanol). The membranes were blocked with 5% (w/v) dry milk in TBS with 0.5% of Tween-20, incubated with primary anti-mouse anti-ERAP1 or anti-βactin Abs in blocking solution overnight at 4 °C, and then with secondary anti-mouse Ab for 1 h at RT (Supplementary Table 3). Secondary Ab detection was performed with Western Lightning ECL Pro (PerkinElmer) and the signal was acquired with Invitrogen iBright CL1500.

### Flow-cytometry analysis

All antibodies used are listed in Supplementary Table 3. For surface staining, cells were incubated with fluorescent labelled Abs in PBS with 2% FBS (FACS buffer) for 20 min on ice. Viability was assessed by staining with Fixable Viability Stain 620 (FVS620) (BD Horizon) or 6-diamidino-2-phenylindole hydrochloride (DAPI, Sigma-Aldrich) (Supplementary Table 3). For cell cycle studies, tumor cells were detached, counted and fixed for 45 min in methanol/acetone 4:1. After centrifugation at 300 g for 5 min, cells were stained with a solution containing 100 µg/mL RNase A and 50 µg/mL propidium iodide (Sigma-Aldrich) overnight in the dark at 4 °C and DNA content was quantified. For the apoptosis assay, Annexin V-FITC Apoptosis Detection Kit (eBioscience) was used according to the manufacturer's protocol. To induce the expression of MHC class I on the cell surface with IFNγ or entinostat, tumor cells were seeded at a density of 30,000 cells for well in a 12-well plate. Cells were treated with mIFNγ (100 U/mL) for 24 h or with entinostat (2 µM) for 48 h. The latter condition was established by treating cells with different concentration (0.5, 1, 2 and 5 µM) of entinostat, and different time points (0, 24, 48, 72 and 96 h). Samples were analyzed on a BD Fortessa X-20 flow cytometer and FlowJo software (Treestar; version 109).

### Migration assay

Migration assay was performed by seeding tumor cells (8000 per well) into the Culture-Insert 2 Well (Ibidi). After cell attachment, a cell-free gap is created in which cell migration can be visualized. At the confluent stage, culture inserts were gently removed creating a 500 µm cell-free gap. Imaging was performed at different time points (0, 24 and 36 h) by LEICA DMi8 microscope (Leica Microsystems). Background was removed with Ilastik software, that uses a machine learning algorithm [31]. The percentage of cell free area was evaluated using a custom CellProfiler pipeline (https://cellprofiler.org).

### 2D and 3D proliferation assays

For 2D proliferation assay, 9464D cells were seeded at the concentration of 2000 cells per well in 96-well flat-bottom plates. The ATP content was determined at different points by the CellTiter-Glo Luminescent Cell Viability assay (Promega). Luminescence was measured with the automatic microplate reader BioTek Synergy H1 (Agilent). 3D tumor spheroids were obtained by seeding 9464D cells into 96-well ultra-low attachment (ULA) U-bottom plates (Corning) at a density of 2000 cells per well. Imaging of tumor spheroids were performed at different time points by LEICA DMi8 microscope. The diameter of the tumor spheroids was measured with the ImageJ software.

### Tumor model and drug treatments

Six to 8-week-old female C57BL/6 black mice were purchased from Charles River Laboratories and housed under pathogen-free conditions in the Plaisant animal facility (Rome, Italy). In vivo experiments were performed in accordance with the 3Rs policy and reviewed and approved by the Italian Ministry of Health (authorization n. 755/2019-PR). 9464D cells (1 × 10^6^) in sterile 1X PBS were injected subcutaneously onto the flank of female C57BL/6 mice. Once tumors reached 80–100 mm^3^ in tumor volume, mice were randomized in groups carrying uniform average tumor load. Each group was treated with vehicle (DMSO), entinostat, anti-PD-1 or the combination (entinostat plus anti-PD-1). For experiments shown in Fig. 3, entinostat was reconstitute in 1% DMSO and administrated at a daily dose of 5 mg/kg via intraperitoneal injection for 14 days. For experiments shown in Fig. 4, to avoid simultaneous intraperitoneal administration of entinostat and anti-PD-1, mice were treated with entinostat (10 mg/Kg, via oral gavage for 21 days), anti-PD-1 Ab (300 mg/mouse, via intraperitoneal injection 3 times per week for 21 days), or a combination of entinostat and anti-PD-1 Ab. Control mice received an equivalent volume of DMSO. For tumor immune infiltrating study, mice were euthanized by CO_2_ inhalation 3 days after the end of drug treatment. Tumor volume was measured twice weekly using a caliper and calculated using the formula: V = [Dx(d)^2^]/2, where D = major tumor axis and d = minor tumor axis, and reported as tumor mass volume (mm^3^, mean ± SD). All experiments involved a minimum of 6 mice per group and were performed at least 2 times, yielding similar results.

### Tissue dissection

Tumor masses were excised from mice, cut into small fragments with scissors and then digested with 325 KU/ml DNAse I (Sigma) and 1 mg/ml collagenase III (Worthington Biochemicals) for 30 min on a shaking platform at room temperature (RT). Tumors were filtered through a 70 μm cell strainer, centrifuged and resuspended to single cells for subsequent procedures. Spleens isolated from tumor-bearing mice were homogenized through a 70 μm strainer, rinsed with RPMI and centrifuged for 10 min at 300 g. Red blood cells were lysed in 1X BD Pharm Lyse (BD Biosciences) for 10 min at RT. Splenocytes were then rinsed twice with 1X PBS and used immediately or cryopreserved in liquid nitrogen according to standard protocols until analysis.

### Co-culture experiments and chemokine analysis

Splenocytes were seeded into round bottom 96-well plates coated with anti-CD3 Ab at density of 0.4 × 10^6^ cells per well in a final volume of 200 μL of complete culture medium supplemented with 1000 U/mL hIL-2 (R&D) and 50 μM 2-mercaptoethanol (Gibco). After 72 h, cells were washed in complete culture medium by centrifugation at 1500 rpm for 5 min at 4 °C and rested overnight in the presence of hIL-2. For tumor cell killing and caspase 3/7 assays, splenocytes were labeled with Cell Tracker Red (Invitrogen) immediately prior to co-culture with tumor cells. For tumor cell killing, caspase 3/7 assay and immune cell activation, tumor cells were seeded into 48-well plates (Falcon) at density of 25,000 cells per well and treated with mIFNγ (100 U/mL) overnight. After 24 h, pre-stimulated splenocytes were added to target cells at the E:T ratio of 10:1. For tumor cell killing assay, after 24 h of co-culture, cells were harvested, resuspended in 250 μL of FACS buffer, and 50μL of CountBright fluorescent counting beads (Thermo Fisher Scientific). Propidium Iodide (PI) (10 μg/mL, Sigma-Aldrich) was added immediately before acquisition by flow cytometry. Live tumor cells were identified as negative for both Cell Tracker Red and PI. The absolute number of live cells was determined using the following formula:

Live cells (cells/μL) = (Cell events/Bead events) x (beads in 50 μL/sample volume).

For the caspase 3/7 assay, co-culture was performed for 7 h in the presence of CellEvent™ Caspase-3/7 Green Detection Reagent (Invitrogen). Multi-fluorescence images were taken at 0 and 7 h with the LEICA DMi8 microscope and the number of caspase 3/7-positive tumor cells was determined with ImageJ software. For the activation assay, the percentage of T cells (CD8^+^ and CD4^+^) and NK cells expressing the activation markers CD25 and CD69, as well as producing granzyme B, IFNγ and TNFα was determined by flow cytometry after 18 h of co-culture. To enhance the detection of cytokine-producing cells, GolgiPlug (BD Biosceince) was added to the co-culture for the last 5 h. For the cell migration assay, tumor cells were seeded into the lower chamber of 6.5 mm Transwell® with 5.0 µm Pore Polycarbonate Membrane Insert (Corning) at density of 150,000 cells for well. The day after, pre-stimulated splenocytes (500,000) were added to target cells into the insert. After 2 h, migrating cells were collected and 800 μl of supernatant was recovered for chemokine analysis using the Proteome Profiler Mouse XL Cytokine Array Kit (R&D Systems) according to the manufacturer’s instructions. The signal was detected using Western Lightning ECL Pro (PerkinElmer) and individual chemokine spots quantified using Image Studio Lite software (version 5.2). To assess the composition of immune cells that migrated through the transwell, cells were stained with specific antibodies. 50 μL of fluorescent CountBright counting beads were added and the immune cell composition was evaluated by flow cytometry.

### Immunohistochemistry and immunofluorescence analyses

Immunohistochemistry (IHC) and multiple immunofluorescence (IF) stainings were performed in 2 μm of formaldehyde-fixed paraffin embedded serial tissue sections as previously described [14, 32]. Antigen retrieval and deparaffination were carried out on a PT-Link (Dako) using EnVision FLEX Target Retrieval Solution Kits at high pH for all stainings. For IHC, following unmasking, slides were subject to the FLEX Peroxidases blocking reagent (Dako) for 10 min, followed by 30 min with 5% PBS/BSA, and then incubated overnight at 4 °C with primary Abs (Supplementary Table 3). This step was followed by incubation with secondary Ab coupled with peroxidase (Dako) for 20 min. Bound peroxidase was detected with diaminobenzidine solution and EnVision FLEX Substrate buffer containing peroxide (Dako). Tissue sections were counterstained with EnVision FLEX hematoxylin (Dako). Iso-type-matched mouse monoclonal Abs (mAb) were used as negative controls. Stained slides were analyzed using an image analysis workstation (Nikon Eclipse E600), scanned using the NanoZoomer S60 Digital slide scanner C13210-01 (Hamamatsu Photonics) and viewed with Hamamatsu Photonics’s image viewer software (NDP.view2 Viewing software U12388–01). For double IF staining, slides were blocked for 1 h with 1% BSA and 5% normal goat serum and then incubated with primary anti-CD8 or anti-NK1.1 Abs, overnight at 4 °C, followed by 1-h incubation with secondary fluorescent Abs. Slides were then stained with anti-granzyme B or anti-IFNγ-CF594 overnight at 4 °C. Granzyme B staining was followed by 1-h incubation with secondary fluorescent Ab. After staining, slides were counterstained with Hoechst (H3570, Invitrogen) for 5 min and cover-slipped with 60% glycerol in PBS. Confocal microscopy imaging was performed by Leica TCS-SP8Xlaser-scanning confocal microscope (Leica Microsystems) equipped with tunable white light laser source, 405 nm diode laser, 3 (PMT) e 2(HyD) internal spectral detector channels. Sequential confocal images were acquired using a HC PLAPO 40 × oil immersion objective (1.30 numerical aperture, Leica Microsystems) with a 1024 × 1024 image format, scan speed 400 Hz. The density of intratumoral CD8^+^ T cells and NK cells expressing granzyme B or IFNγ was recorded by two blinded examiners as the number of positive cells per unit tissue surface area (mm^2^). The mean of the positive cells detected in 5 fields for each sample was used in the statistical analysis.

### Immunopeptidomics analysis

Abs specific to MHC class I molecules H-2K^b^ and H-2D^b^ were produced in house from the supernatants of Y-3 [33] and 28.14.S hybridomas [34]. Secreted mAbs were harvested from the culture medium and purified with Protein A Sepharose (PAS, CaptivA®, Repligen, USA) using a Profinia purification system (BioRad). Small scale immunoaffinity purifications were performed on sgCTR3- and sgE-1-transduced 9464D cells of pellet size 6 × 10^7^ cells per condition as previously described [35]. Briefly, cell pellets were lysed with 300 µL of lysis buffer (0.5% IGEPAL, 50 mM Tris [pH 8.0], 150 mM NaCl and 1X protease inhibitor tablet [cOmpleteTM Protease Inhibitor Cocktail Tablet; Roche Molecular Biochemicals, Switzerland]), mixed gently and incubated on a roller at 4 °C for 1 h. Samples were lysed at 4 °C for 45 min and samples were centrifuged at 3724 g, 10 min. Peptide-MHC (pMHC) class I complexes were pulled down in a sequential manner (Y-3 first followed by 28.14.S). To remove contaminants such as detergent, salt, and nonspecific binders, all columns were washed with 5 column volumes of 1X PBS. Peptides of the pMHC class I complexes were eluted using 300 µL of 10% acetic acid. To separate the proteinaceous material from peptides, the eluate was passed through a 5 kDa molecular weight cut off filter (Amicon, Sigma-Aldrich, USA) and centrifuged at 16,060 g for 30 min at RT. The samples were dried down, and reconstituted in buffer A (2% Acetonitrile [ACN] and 0.1% formic acid [FA]).

### Analysis of MHC class I bound peptides in 9464D samples using an Evosep One—Bruker Tims TOF Pro 2 LC–MS system

A mixture of 11 indexed retention time (iRT) peptides was spiked into the 2 samples and the samples were loaded onto Puretips (Evosep Biosystems) as per manufacturer’s instructions. Evosep one liquid chromatography (LC) system was used to acquire the samples on the Bruker TimsTOF Pro2 instrument. Samples were eluted and separated on an Aurora Elite column (IonOpticks, 15 cm x 75um × 1.7 um, 120 A pore size) using the Zoom Whisper 20 SPD (flow rate of 200nL/min, 68 min long gradient with 100% buffer B wash at the end) at 50 °C. Mobile phases A and B consisted of 0.1% FA in 2% ACN and 0.1% FA in ACN, respectively. The peptides eluted from the column were analysed using a hybrid trapped ion mobility-quadrupole time of flight mass spectrometer (Bruker timsTOF Pro 2, Bruker Daltonics). Data dependent acquisition was performed with the following settings: m/z range: 100-1700mz, capillary voltage:1600 V, Target intensity of 30,000, TIMS ramp of 0.60 to 1.60 Vs/cm 2 for 166 ms.

### Data analysis

The raw data files obtained from the Bruker TimsTOF Pro2 were analysed using Peaks Online software (ver 11, Bioinformatics Solutions Inc, [36]) and searched against the mouse proteome (Uniprot 12/08/2024; 25,601 entries). The following search parameters were used for the samples: error tolerance of 15 ppm using monoisotopic mass for precursor ions and 0.05 Da tolerance for fragment ions. Enzyme used was set to none with following variable modifications: oxidation at Met (M), deamidation at Asp (D) and Gln (Q). The false discovery rate (FDR) was estimated using a decoy fusion method for the samples [36] and all datasets were analysed using a 5% FDR cut off. The predicted binding affinity of the peptides to the MHC-I molecules was estimated using the NetMHC-4.0 tool [37]. Venn diagrams were made using the ggvenn package and MHC motifs were visualized using the ggseqlogo package [38], under R version 4.4.1 [39] (https://www.R-project.org/). The mass spectrometry proteomics data have been deposited to the ProteomeXchange Consortium via the PRIDE partner repository with the dataset identifier PXD055268 and 10.6019/PXD055268.

### Statistical analysis

GraphPad prism 8.0.2 software was used to calculate significance between the samples. Two-tailed Student's t-test was used to compare the means of two groups. One-way ANOVA was used to compare the means of three or more groups. Data for mice survival were presented using Kaplan–Meier survival curves and log-rank test was performed to determine statistical significance between treatment groups. Unless specifically stated, all data are representative of > 3 separate experiments. Error bars represent SD. Value of p ≤ 0.05 was considered to be statistically significant.

## Results

### Downregulation of ERAP1 does not affect the surface expression of MHC class I molecules in 9464D cells

**Fig. 1:**
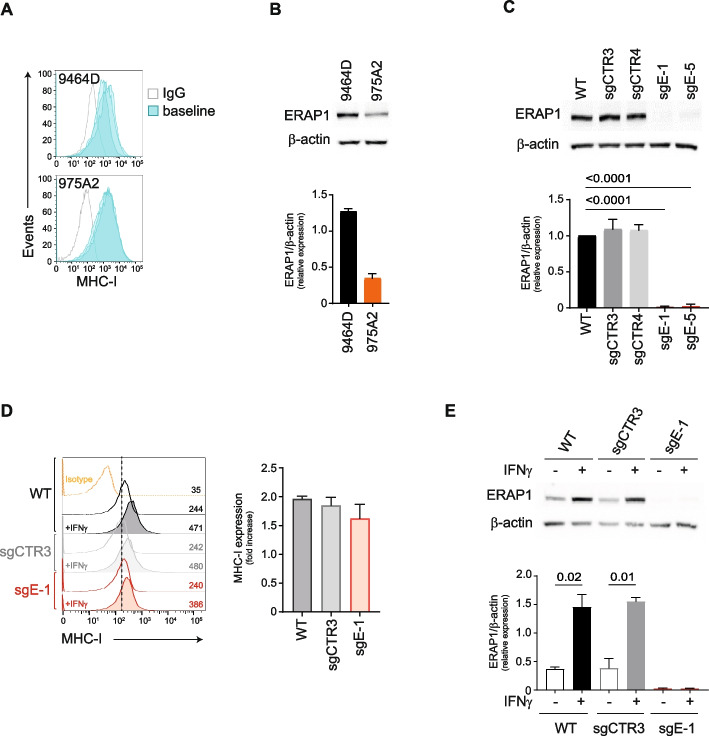
Inhibition of ERAP1 does not affect the surface expression of MHC class I molecules of 9464D cells. **A** Representative flow-cytometry histograms of MHC class I expression in 200 mm^3^-size 9464D and 975A2 tumors (*n* = 3) grown subcutaneously in C57BL/6 mice. **B** Representative immunoblotting analysis of ERAP1 expression in 9464D and 975A2 NB cell lines. Densitometric analysis of β-actin-normalized ERAP1 expression from three independent experiments is shown below. **C** Representative immunoblotting analysis of ERAP1 expression in 9464D cells untreated or infected with lentiviruses carrying non-targeting sgRNAs (sgCTR3 and sgCTR4) or sgRNAs targeting exon 1 or exon 5 (sgE-1 and sgE-5) of the *ERAP1* gene. Densitometric analysis of β-actin-normalized ERAP1 expression from three independent experiments is shown below. **D** Representative flow-cytometry histograms of MHC class I expression in the indicated cell lines. Isotype-matched negative control Ab is shown as yellow histogram. Bars represent the increase in mean fluorescence intensity (MFI) of MHC class I expression in IFNγ-stimulated compared to unstimulated cells. **E** Representative immunoblotting analysis of ERAP1 expression in the indicated cells untreated or treated with IFNγ. Densitometric analysis of β-actin-normalized ERAP1 expression from three independent experiments is shown below. Levels of significance for comparison between samples were determined by ANOVA and two-tailed Student’s t test. Statistically significant P values are shown

To investigate the potential impact of ERAP1 modulation on the immune response to NB, we analyzed the expression of surface MHC class I molecules and ERAP1 in two transplantable NB mouse models, 9464D and 975A2, derived from spontaneous tumors arising in TH-MYCN transgenic mice [28, 40]. Similar to high-risk human NBs, both tumor models express low levels of MHC class I on the cell surface (Fig. 1A). This is a reversible phenomenon in the NB that results from epigenetic and/or transcriptional and post-transcriptional regulation [15, 16]. Consistently, treatment with IFNγ restored MHC class I expression in both cell lines, albeit at different levels (Supplementary Figure S1). Western blot analysis revealed a higher expression of ERAP1 in the 9464D cell line (Fig. 1B), prompting us to select this tumor model for further experiments.

We used CRISPR/Cas9-mediated gene editing toKO the *ERAP1* gene in the 9464D cell line. We cloned two single guide RNAs (sgRNAs) targeting the first and the fifth exons of the *ERAP1* gene (sgE-1 and sgE-5, respectively) (Supplementary Figure S2A) into the 3rd-generation lentiCRISPRv2GFP vector, which constitutively expresses Cas9 together with GFP (Supplementary Figure S2B). As control, cells were transduced with two independent non-targeted sgRNAs, i.e., sgCTR3 and sgCTR4. Western blot analysis confirmed the loss of ERAP1 protein expression in sgE-1 and sgE-5 cells (Fig. 1C). To assess the efficiency of *ERAP1* gene silencing, DNA sequences spanning sgRNA target sites were sequenced and analyzed using the two web-based bioinformatic tools CRISP-ID and TIDE [29, 30] (Supplementary Figure S2C). Cas9-induced mutations in the predicted cleavage sites upstream of the PAM sequence in both sgE-1 and sgE-5 cells (Supplementary Figure S2C). Deconvolution of the sequence trace data showed that 90% of sgE-1 cells had an indel in exon 1, corresponding to a deletion of one base in 60.3% of cases, whereas 94.6% of sgE-5 cells had an indel in exon 5, corresponding to the insertion of one base in 60.4% of cases (Supplementary Figure S2C).

As loss of ERAP1 has been associated with reduced cell proliferation in some tumor models [41], we evaluated the proliferation rate of ERAP1 KO and control cells under 2D and 3D growth conditions as well as cell cycle, apoptosis and cell migration. Loss of ERAP1 did not affect any of the properties tested in 9464D cells (Supplementary Figure S3). Since the sgE-1 and sgE-5 cell lines show overlapping data, we focused on sgE-1 and sgCTR-3 cells as control in subsequent experiments.

Contrary to the other APP components, inhibition of ERAP1 only marginally affects the surface expression of MHC class I molecules [42]. Consistently, we found no significant change in the expression of MHC class I on sgE-1 cells compared to sgCTR3 cells (Fig. 1D and Supplementary Figure S4A). Of note, the expression of MHC class I molecules was induced by IFNγ in all cells tested to a similar level (Fig. 1D). Western blot analysis showed that IFNγ treatment induced ERAP1 expression in control cells (both wild-type and sgCTR3), but not in sgE-1 cells, further confirming the lack of *ERAP1* gene expression (Fig. 1E). Inhibition of ERAP1 did not affect the induction of the other components of the APP pathway (tapasin [TAPBP] and beta-2 microglobulin (β2m) nor of the IFNγ signaling pathway (IRF1, IRF2 and STAT1) after stimulation with IFNγ (Supplementary Figure S4B).

Altogether, these data indicate that ERAP1 inhibition does not alter either basal or IFNγ-induced expression of APP components, including MHC class I molecules on the surface of 9464D cells.

### Inhibition of ERAP1 renders 9464D cells more susceptible to lysis by immune cells

**Fig. 2:**
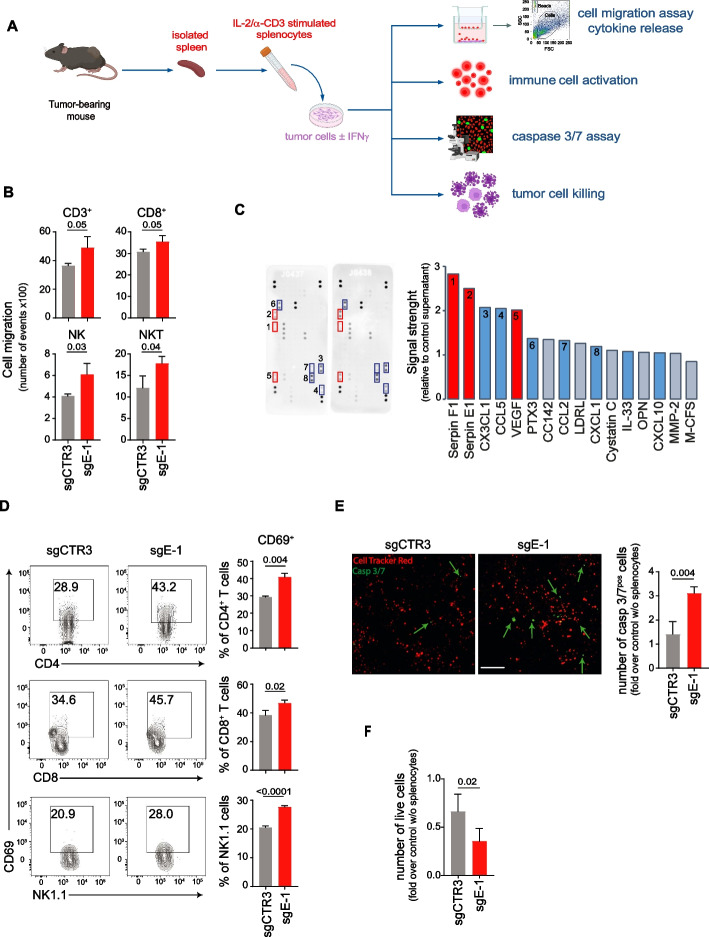
Inhibition of ERAP1 renders 9464D cells more susceptible to lysis by immune cells. A Experimental scheme. Tumor cells untreated or treated with IFNγ were co-cultured with pre-stimulated syngeneic splenocytes derived from tumor-bearing mice. **B** Quantification of immune cells migrated through a trans-well to tumor cells. Bars indicate the total number of migrated cells. **C** Chemokine expression in sgCTR3 and sgE-1 cell lysates by protein array. Relative chemokine expression based on densitometric analysis is shown on the right. **D** Representative flow-cytometry analyses of CD69 expression by CD4^+^ T cells, CD8^+^ T cells and NK cells from splenocytes co-cultured with IFNγ-treated tumor cells for 18 h. Bars represent the % of immune cells expressing the indicated markers. **E** Representative multi-fluorescence images of IFNγ-treated tumor cells co-cultured 7 h with red-labeled splenocytes in the presence of caspase 3/7 Green Detection Reagent, shown at original magnification × 20, scale bar 75 μm. Caspase 3/7-positive tumor cells are indicated by green arrows. Quantitative analysis of the caspase 3/7-positive tumor cells from at least 8 fields for each of two independent experiments is shown. **F** Live IFNγ-treated tumor cells after 24 h of co-culture with splenocytes. Data are normalized to the number of live tumor cells without splenocytes. Levels of significance for comparison between samples were determined by two-tailed Student’s t test. Statistically significant *P* values are shown

We and others have previously shown that the immunopeptidome change generated by *ERAP1* silencing is able to influence the anti-tumor responses mediated by T cells and NK cells [23–26]. To evaluate the potential of sgE-1 cells to induce activation and recruitment of immune cells, we studied IFNγ-treated or untreated sgCTR3 and sgE-1 cells cocultured with splenocytes derived from wild-type 9464D-bearing mice (Fig. 2A).

We found that significantly more CD3^+^ T cells, CD8^+^ T cells, NK cells and NKT cells were attracted to sgE-1 cells than to sgCTR3 cells after 2 h of co-culture (Fig. 2B). A protein array of the supernatant of the coculture of splenocytes and sgE-1 cells showed more than twofold increases in chemokines involved in immune cell recruitment, such as CX3CL1 and CCL5, compared with that of cocultures of splenocytes and sgCTR3 cells (Fig. 2C). PTX3 and CCL2 also increased more than 1.3-fold (Fig. 2C). We also observed more activated CD8^+^ T cells and NK cells after 18 h of coculture with sgE-1 cells than control cells, with significantly higher expression of surface and intracellular activation markers such as CD69 and granzyme B, TNFα and/or IFNγ, respectively (Fig. 2D, Supplementary Figure S5A and S5B). CD69 and CD25 were also higher expressed in CD4^+^ T cells when cocultured with sgE-1 cells (Fig. 2D, Supplementary Figure S5C). Furthermore, we detected an increase of caspase 3/7-positive cells in sgE-1 compared to sgCTR3 after 7 h of co-culture (1.4 ± 0.2 and 3.1 ± 0.1 fold, respectively, compared to the condition without splenocytes) (Fig. 2E). Consistently, we found fewer live sgE-1 cells than control cells after 24 h of coculture (0.7 ± 0.09 and 0.3 ± 0.06 of live cells, respectively) (Fig. 2F).

Overall, these data suggest that loss of ERAP1 expression makes tumor cells more susceptible to killing by immune cells, as a result of increased release of inflammatory cytokines and recruitment and activation of immune effector cells.

### Lack of ERAP1 is not sufficient to control the in vivo growth of 9464D cells

**Fig. 3:**
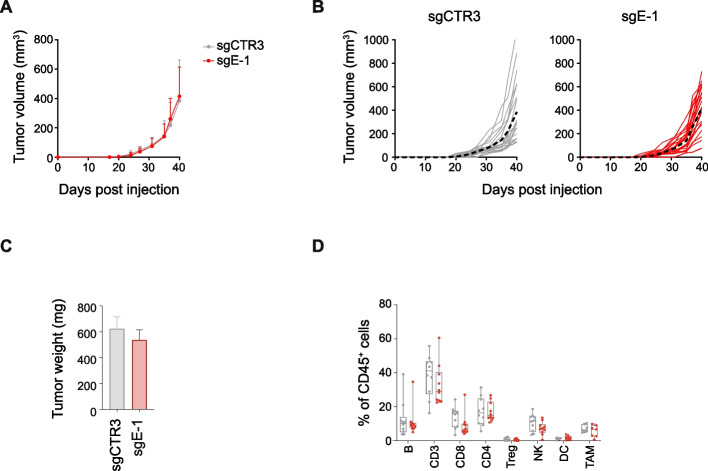
ERAP1 inhibition does not alter the growth and immune landscape of 9464D tumors. **A, B **Tumor growth of sgCTR3 and sgE-1 cells injected subcutaneously in C57BL/6 mice. Growth curves of groups (**A**) and single mice (**B) **are shown**. ** In **B**, the average growth for each group is indicated with a black dotted line. **C** Weight of explanted tumors at 40 days post injection. **D**, Flow-cytometry analysis of the immune content in explanted tumors at day 40 post injection (n ≥ 10 for each group). Levels of significance for comparison between samples were determined by two-tailed Student’s t test

We next evaluated the effect of ERAP1 deficiency on tumor growth in vivo. sgE-1 and sgCTR3 cells were engrafted into the left flank of syngeneic C57BL/6 mice and tumor size was monitored over time. We observed that the absence of ERAP1 was not sufficient to control the growth of 9464D tumors in syngeneic mice (Fig. 3A-C). We used multi-color flow-cytometry panels to quantify the relative frequency of different immune cell populations in tumor masses of 200–300 mm^3^ (Supplementary Figure S6). We observed no difference in the immune infiltrate between sgE-1 and sgCTR3 tumors (Fig. 3D). We hypothesized that the low level of MHC class I expression in these tumors might mask the effect mediated by loss of ERAP1.

### The lack of ERAP1 affects the expression of entinostat-induced MHC class I molecules in 9464D cells

**Fig. 4:**
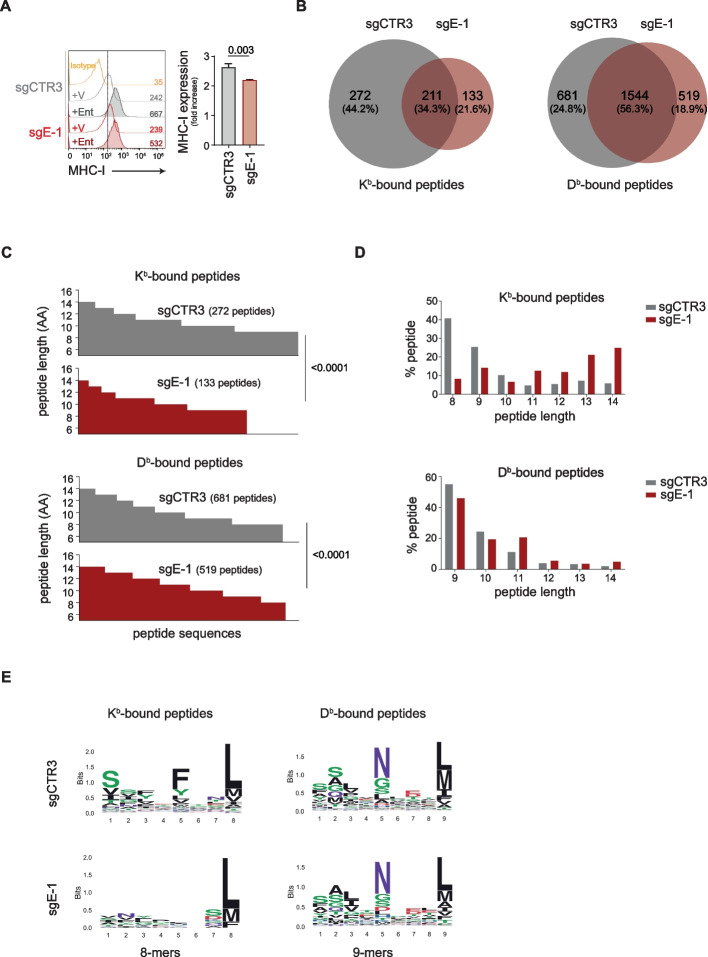
Inhibition of ERAP1 affects both the surface expression of MHC class I molecules and the immunopeptidome of 9464D cells treated with entinostat. **A **Representative flow-cytometry histograms of MHC class I expression in the indicated cell lines. Isotype-matched negative control Ab is shown as yellow histogram. Bars represent the increase in MFI of MHC class I expression in entinostat- (Ent) stimulated compared to unstimulated tumor cells. **B** Venn diagrams showing the number of unique and shared H-2K^b^- and H-2D^b^-bound peptides between sgCTR3 and sgE-1 cells. **C **and **D** The number (**B**) and the percentage (**C**) of peptides bound to H-2K^b^- and H-2D^b^of sgCTR3 and sgE-1 cells are plotted according to their amino acid length. **E**, Logo representation of unique H-2K^b^- and H-2D^b^-bound peptide sequences in sgCTR3 and sgE-1 cells analyzed independently according to their lengths shown on the x-axis. The height of each column is proportional to the degree of amino acid conservation and the height of each letter composing the column is proportional to its frequency at the given position. Numbers between parentheses indicate the number of peptide sequences analyzed. Amino acids are colored as follows: acidic (red), basic (blue), hydrophobic (black), neutral (purple) and polar (green)

To test whether the effect of ERAP1 inhibition on 9464D cells is masked by the low expression levels of MHC class I molecules, we evaluated the efficacy of entinostat in upregulating the expression of MHC class I molecules in our tumor model. 9464D cells were treated for 24, 48 or 72 h with increasing concentrations of entinostat (Supplementary Figure S7A-C). The 48-h treatment with 2μM entinostat was able to induce at least a twofold increase in the surface expression of MHC class I molecules and maintain the viability of 9464D cells (Supplementary Figure S7A-C). This condition was used for subsequent experiments. Interestingly, entinostat was able to induce the expression of MHC class I molecules in both sgCTR3 and sgE-1 cells (2.6 ± 0.07 and 2.2 ± 0.01, respectively), but to a significantly lesser extent in the absence of ERAP1 (Fig. 4A). Similarly, the nonclassical MHC class I molecule Qa-1b, the functional homolog of HLA-E in humans, was induced by entinostat in both sgCTR3 and sgE-1 cells, but to a lesser extent in sgE-1 cells (1.5 ± 0.05 and 1.3 ± 0.02, respectively) suggesting an involvement of ERAP1 in the generation of peptide ligands of Qa-1b molecules (Supplementary Figure S7D). In contrast, RAE1 and CD86, an NK cell receptor ligand and a costimulatory molecule, respectively, which are known not to bind peptides, were both induced by entinostat in sgE-1 and sgCTR3 cells in the same way (Supplementary Figure S7D).

### Inhibition of ERAP1 leads to a change in the immunopeptidome of 9464D cells

To examine the impact of ERAP1 activity in shaping the immunopeptidome, we perfomed mass spectrometry of entinostat-treated sgE-1 and sgCTR3 cells. After affinity purification, peptides were eluted from H-2K^b^ and H-2D^b^ and analysed. A total of 3419 (8–14 amino acids) and 7776 (9–14 amino acids) peptides were identified from H-2K^b^ or H-2D^b^ molecules, respectively (Supplementary Tables 4 and 5). We used the NetMHC-4.0 tool [37] to predict the binding affinity of the peptides to H-2K^b^ and H-2D^b^ molecules. Only peptides with a predicted binding rank ≤ 2 were considered for further analysis. To assess the qualitative differences in the immunopeptidome in the presence or absence of ERAP1, we focused on unique binders of each MHC class I molecule (405 and 1200 peptides for H-2K^b^ or H-2D^b^, respectively) (Fig. 4B, Supplementary Tables 4 and 5). The sequenced peptides were grouped according to length (Fig. 4C and 4D). Among the 272 H-2K^b^ and 681 H-2D^b^-bound peptides in sgCTR3 cells, 40% and 55% were, respectively, 8-mers and 9-mers in agreement with previous findings [43] (Fig. 4C and 4D). The frequency of canonical peptide lengths were significantly altered in the absence of ERAP1 (Fig. 4C and 4D). For both H-2K^b^ and H-2D^b^ alleles in sgE-1 cells, there was a decrease in the recovery of canonical length peptides (8% and 45%, respectively) and a concomitant increase in longer peptides. Peptides ≥ 11 residues bound to H-2Kb increased from 8 to 19%. A 9% increase was observed for 11-mers for H-2D^b^. In contrast, these differences in peptide length distribution were not observed in the peptides shared between sgE-1 and sgCTR3 cells (Supplementary Figure S8), further supporting previous findings on the uniqueness of the immunopeptidome in the absence of ERAP1 [44].

To further characterize the changes in the immunopeptidome, we analysed the peptide sequences for the distribution of conserved amino acids in sgCTR3 versus sgE-1 cells. We generated the sequence logos using the ggseqlogo package [38]. The most conserved and frequent residues in H-2K^b^-bound canonical 8 mers were the aromatic phenylalanine (F) or tyrosine (Y) at p5 and an aliphatic residue (L, M, I, or V) at the C terminus [43, 45]. For H-2D^b^-bound canonical 9 mers, the conserved residues were the asparagine (N) at p5 position and an aliphatic residue (L, I, or M) at the C terminus [43, 45]. Notably, these consensus motifs were conserved in the canonical peptide length recovered from H-2K^b^ (8 mers) and H-2D^b^ (9 mers) in sgE-1 cells, with the exception of the consensus motif of the P5 position in H-2K^b^-bound peptides, which was completely lost (Fig. 4E).

Overall, the mass-spectrometry analysis clearly showed that the canonical lengths and composition of the immunopeptidome presented by both H-2K^b^ and H-2D^b^ had a strong dependence on ERAP1, with a unique immunopeptidome found in the ERAP-1 KO cells.

### Lack of ERAP1 during entinostat treatment reprograms the tumor immune microenvironment to delay tumor progression

**Fig. 5:**
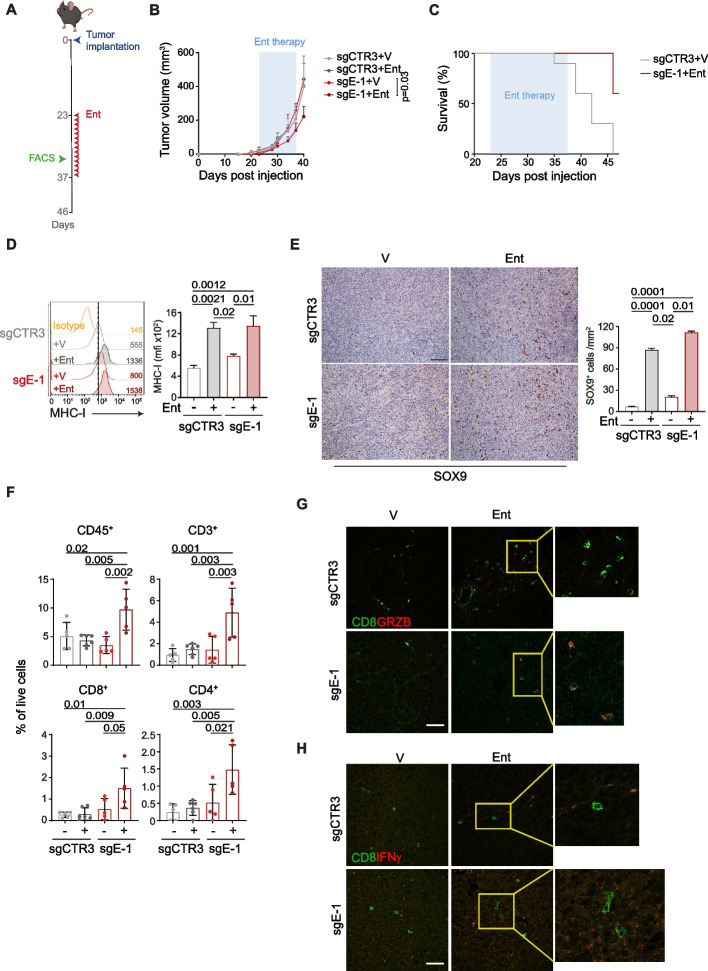
Inhibition of ERAP1 in combination with entinostat treatment delays the growth of 9464D tumors and reshapes the intratumoral immune infiltrate. **A** Schematic representation of the entinostat treatment and timing of tumor immune infiltrate analysis. **B** Tumor growth of sgCTR3 and sgE-1 cells injected subcutaneously in C57BL/6 mice and treated as indicated. Significance at day 17 after the start of treatment. Levels of significance for comparison between samples were determined by ANOVA. Statistically significant P values are shown. **C** Survival analysis of the indicated experimental groups. Levels of significance for comparison between samples were determined by Log-rank test. Statistically significant P values are shown. **D** Representative flow-cytometry histograms of MHC class I expression in the explanted tumors at day 10 after the start of treatment (n ≥ 5 for each group). Isotype-matched negative control Ab is shown as yellow histogram. Bars represent the increase in MFI of MHC class I expression in entinostat- (Ent) stimulated compared to unstimulated tumor cells. **E** Representative examples of SOX9 staining in the explanted tumors at day 10 after the start of treatment (n ≥ 5 for each group). Nuclei were counterstained with hematoxylin (blue). A number of SOX9 positive cells are indicated by brown arrows. Original magnifications, × 20. Scale bars, 30 μm. Quantitative analysis of SOX9 expressing cells from *n* = 3 biologically independent highly infiltrated NBs is shown on the right. Plotted as mean ± S.D. and analyzed by Kruskal–Wallis test to generate two-tailed P values. **F** Flow-cytometry analysis of the immune content in the same tumors analysed in E. Levels of significance for comparison between samples were determined by ANOVA and Log-rank test. **G** Representative multiple immunofluorescence staining of the same tumors analysed in E and F for CD8^+^ T cells (green) expressing granzyme B (red) or IFNγ (red) shown at magnification 40 × , scale bar 30 μm. Images with nuclei (Hoechst) are shown on the left of each panel. The yellow rectangles are higly magnified on the right-hand panels. V, vehicle control; Ent, entinostat. Statistically significant P values are shown

To evaluate the antitumor efficacy of entinostat in combination with ERAP1 silencing in vivo, we subcutaneously implanted sgCTR3 and sgE-1 cells into the flank of C57BL/6 mice. When tumors reached 50–80 mm^3^, mice were randomized and treated with vehicle (DMSO) or entinostat (5 mg/kg intraperitoneally) [46] (Fig. 5A). Interestingly, entinostat treatment significantly inhibited the progression of sgE-1 tumors, but not sgCTR3 tumors, which instead grew similarly to vehicle-treated sgCTR3 and sgE-1 tumors (Fig. 5B and Supplementary Figure S9A). The reduction in tumor progression was also associated with a significant increase in host survival (Fig. 5C and Supplementary Figure S9B). At 46 days post injection all control mice were dead, whereas 60% of sgE-1 tumor-bearing mice were alive (Fig. 5C and Supplementary Figure S9B), suggesting that loss of ERAP1 renders 9464D tumors responsive to entinostat treatment. Similar to what was observed in vitro, entinostat treatment was able to significantly increase surface expression of MHC class I molecules in both entinostat-treated tumors (2.38 and 1.67, for sgCTR3 and sgE-1, respectively) (Fig. 5D). Entinostat treatment is also known to convert NB cells from the adrenergic (ADR) to the more immunogenic mesenchymal (MES) phenotype [19, 47]. We therefore tested whether the increased immunogenicity was accompanied by a switch of tumor cells to the MES phenotype. IHC analysis revealed that entinostat-treated tumors expressed higher levels of the MES marker SOX9 regardless of ERAP1 expression (Fig. 5E). To evaluate the antitumor efficacy of entinostat in vivo, we quantified the relative frequency of different immune cell populations in tumors harvested after 10 days of treatment. We observed an increase in the immune infiltrate of entinostat-treated sgE-1 tumors (Fig. 5F). We found a higher number of TILs, particularly CD8^+^ and CD4^+^ T-cell subsets, compared to controls (Fig. 5F). NK cells were also increased in sgE-1 tumors, although not significantly (Supplementary Figure S9C). The IF analysis confirmed the FACS data and also showed an enrichment of CD8^+^ T cells expressing IFNγ and granzyme B as well as NK cells expressing IFNγ in entinostat-treated sgE-1 tumors compared to the other conditions analysed (Fig. 5G and 5H, Supplementary Figure S10).

Collectively, these results demonstrate that loss of ERAP1 in combination with entinostat treatment promotes an inflamed T-cell phenotype that results in NB growth control.

### Lack of *ERAP1* in combination with entinostat and PD-1 blockade control tumor growth and increase host survival

**Fig. 6:**
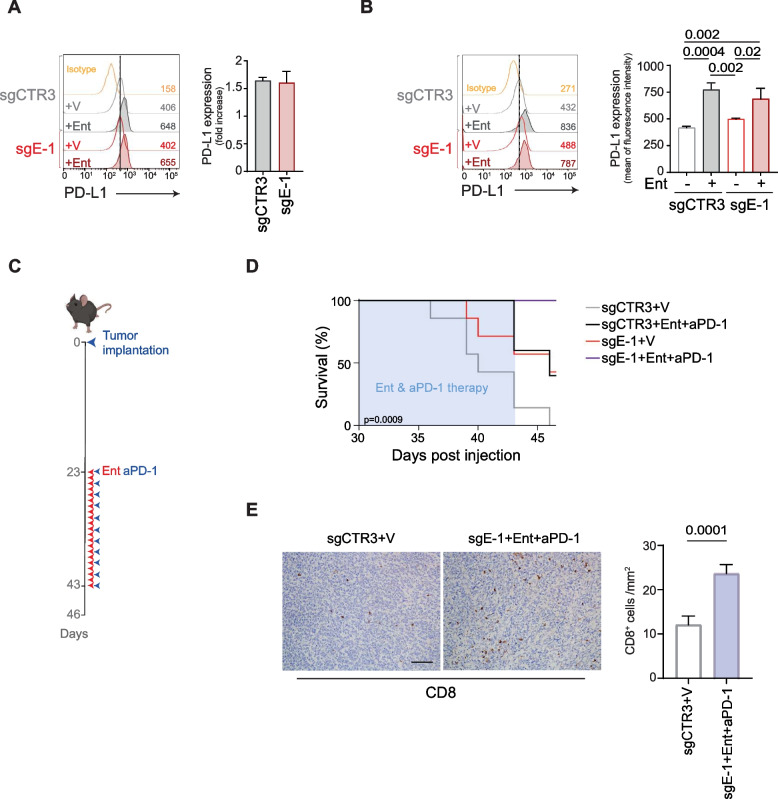
ERAP1 inhibition in combination with entinostat and PD-1 blockade delays the growth of 9464D tumors. **A** and **B** Representative flow-cytometry histograms of PD-L1 expression in the indicated cell lines (**A**) and explanted tumors (**B**) at day 10 after the start of treatment (n ≥ 5 for each group). Isotype-matched negative control Ab is shown as yellow histogram. Bars represent the increase in MFI of PD-L1 expression in entinostat- (Ent) stimulated compared to vehicle-treated tumor cells. **C** Schematic representation of the combined entinostat and PD-1 treatment. **D** Survival analysis of the indicated experimental groups. Levels of significance for comparison between samples were determined by ANOVA, two-tailed Student’s t test and Log-rank test. **E** Representative examples of CD8 staining in the explanted tumors at day 10 after the start of treatment (n ≥ 7 for each group). Nuclei were counterstained with hematoxylin (blue). Original magnifications, × 20. Scale bars, 30 μm. Quantitative analysis of CD8 expressing cells from *n* = 3 biologically independent highly infiltrated NBs is shown on the right. Plotted as mean ± S.D. and analyzed by Kruskal–Wallis test to generate two-tailed P values. V, vehicle control; Ent, entinostat; aPD-1, anti-PD-1 Ab. Statistically significant *P* values are shown

Entinostat is also known to induce PD-L1 expression in several tumor models [48–50]. Indeed, we observed increased PD-L1 expression in entinostat-treated 9464D both in vitro and in vivo, regardless of ERAP1 expression (Fig. 6A and B). Since high levels of PD-L1 expression are predictive of the response to immune checkpoint blockade, we tested if entinostat could increase the efficacy of PD-1 blockade. sgE-1 and sgCTR3 cells were injected subcutaneously into C57BL/6 and once tumors reached 100 mm^3^, mice were randomized into 4 treatment groups: vehicle plus control IgG, entinostat, anti-PD-1, and entinostat plus anti-PD-1 (Fig. 6C). To avoid a possible reduction in the efficacy of entinostat and anti-PD-1 due to intraperitoneal administration of both drugs, entinostat was administrated by oral gavage. This different mode of administration did not change the efficacy of entinostat, which was always more effective in tumors lacking ERAP1 (Supplementary Figure S11A). Treatment with anti-PD-1 alone was ineffective regardless of ERAP1 expression (Supplementary Figure S11B). The combination of entinostat with anti-PD-1 was more effective in sgE-1 tumors than any other condition studied in terms of survival analysis (Fig. 6D and Supplementary Figure S11C and S11D). Interestingly, combined therapy (entinostat plus anti-PD-1) did not improved the survival of sgCTR3-bearing mice compared to treatment with entinostat alone (40% of mice alive in both cases) (Supplementary Figure S11C), whereas it brought substantial benefit to sgE-1 tumor-bearing mice (Supplementary Figure S11D). Indeed, at 46 days post injection, all sgE-1 tumor-bearing mice treated with combination therapy were alive compared to 57% of those treated with entinostat alone (Supplementary Figure S11D). Moreover, IHC analysis revealed that sgE-1 tumors treated with the combination therapy were infiltrated by significantly more CD8^+^ T cells than control tumors (Fig. 6E).

Altogether, these data demonstrate that the lack of ERAP1 enhances the efficacy of combination therapy with entinostat and anti-PD-1 in a tumor model such as 9464D, that is basically unresponsive to PD-1 blockade.

## Discussion

Low TMB and low expression of MHC class I molecules prevent NB from presenting a sufficient repertoire of tumor antigens to induce cytotoxic CD8^+^ T-cell mediated anti-tumor responses [51]. Here we demonstrate that targeting ERAP1 and treatment with the HDACi entinostat curbed NB growth in vivo, making tumors more infiltrated by CD8^+^ T-cells and responsive to PD-1 blockade.

We hypothesize that ERAP1 inhibition, by expanding the repertoire of neoantigens presented on the surface of tumor cells, may function as a surrogate for the low TMB. Alterations in the APP pathway can potentially be exploited to increase the repertoire of tumor antigens presented by MHC class I molecules able of eliciting an efficient immune response [20]. The presentation of these "altered self" peptides is a phenomenon independent of the TMB. This class of antigens represents an interesting category as they are potentially shared by several tumors. A well-characterized example of “altered self” antigens is represented by T-cell epitopes associated with altered peptide processing (TEIPP), a class of non-mutational tumor antigens that arise specifically in transporter associated with antigen processing- (TAP) deficient tumors and efficiently induce a functional antitumor CD8^+^ T cell response in these tumors, but not against TAP-expressing counterparts on healthy tissues [52–55]. Inhibition of ERAP1 is presumed to potentially affect tumor immunopeptidome by a similar mechanism. Several studies have shown that, in the absence of ERAP1, cells exhibit new immunogenic epitopes that are normally destroyed by ERAP1, whereas peptides normally generated by the enzyme are missing [24, 27, 56]. Recently, Leishman and colleagues reported that inhibition of ERAP1 in “hot” tumor models, such as melanoma and colorectal cancer, results in the generation of new epitopes derived from several cancer-associated proteins [57]. Inhibition of ERAP1 in combination with PD-1 blockade results in increased tumor-specific immune response and T-cell receptor (TCR) repertoire diversity in several syngeneic tumor models [57]. This is in line with a study reporting that inhibition of ERAP1 sensitizes the transplantable 4T1 breast cancer cell model to anti-PD-1 immunotherapy [53]. Moreover, two other in vivo genome-wide CRISPR/Cas9 screening studies showed that deletion of APP-related genes in two different cancer models is associated with increased sensitivity to ICI-based immunotherapy, in which ERAP1, calreticulin and tapasin were the most relevant molecules in melanoma, while β2m was in renal carcinoma [58, 59]. Furthermore, a strong association between tumor immunogenic antigen load and TCR diversity was observed in TILs in human cancers [60]. A diverse TCR repertoire is able to offer greater opportunities for tumor neoantigen recognition. Indeed, high TCR diversity prior to ICI therapy has been positively correlated with clinical response in several solid tumors [61].

Herein, we found that ERAP1 inhibition alone has no effect on the in vitro and in vivo growth of the 9464D transplantable mouse model which closely recapitulates the molecular and biological features of high-risk NBs [28, 62–65]. In contrast, the effect was evident in the growth of ERAP1-inhibited tumors, both in vitro and in vivo, in the presence of adequate surface expression of MHC class I molecules induced by stimulation with IFNγ or entinostat that highlighted the pronounced change in the immunopeptidome resulting from modulation of ERAP1. Treatment of ERAP1-expressing tumors with entinostat, while inducing the expression of MHC class I molecules, had no effect on tumor growth. This is consistent with the results obtained in several clinical trials. The phase I clinical trial NCT02780804, designed to test entinostat as a single agent in pediatric patients with relapsed or refractory solid tumors, revealed that although the treatment was well tolerated, all patients showed disease progression within the first two cycles, with the exception of one patient with stable disease [66]. Other studies have shown that treatment with entinostat improves the response to PD-1 blockade in several tumors [67–72]. The combination of entinostat with anti-PD-1 showed robust in vivo antitumor activity in murine bladder tumors, promoting long-term immunological memory [73]. However, its efficacy was reduced in tumors with a lower TMB or low MHC class I expression, suggesting that the anti-tumor activity of entinostat is mainly in immunogenic tumors [73]. In support of this observation, three clinical trials (NCT01928576, NCT02437136, NCT02697630) report that entinostat was successfully administrated in combination with anti-PD-1 antibodies (pembrolizumab or nivolumab) in patients with high TMB that had progressed on or after PD-1/PD-L1 inhibitors such metastatic non-small-cell lung cancer and uveal melanoma [74–76]. In contrast, the combination of entinostat plus anti-PD-L1 antibodies (avelumab or atezolizumab) showed no clinical benefit compared to ICI alone in patients with low TMB, such as epithelial ovarian cancer, triple-negative breast cancer, and myelodysplastic syndrome (NCT02915523, NCT02708680, NCT02936752) [77–79]. This is not surprising given the correlation between TMB and objective response rate to anti-PD-1, which may explain the observed differences in response between cancer types, suggesting that the therapeutic activity of entinostat relies on relevant neoantigen load [80, 81].

In conclusion, we explored a non-toxic approach based on ERAP1 inhibition and entinostat treatment to increase the immunogenicity of NB by making tumor responsive to PD-1 blockade. Our hypothesis is that ERAP1 inhibition, on the one hand, and induction of MHC class I expression, on the other hand, may counteract the low TMB of NB and result in the generation and presentation of new neoantigen repertoire. This increased “immune visibility” may result in the control of tumor growth and increased T-cell infiltration allowing PD-1 blockade to reinvigorate the anti-tumor immune response, thereby further prolonging host survival.

## Supplementary Information

Supplementary Material 1.

Supplementary Material 2.

Supplementary Material 3.

Supplementary Material 4.

Supplementary Material 5.

Supplementary Material 6.

Supplementary Material 7.

Supplementary Material 8.

Supplementary Material 9.

Supplementary Material 10.

Supplementary Material 11.

Supplementary Material 12.

Supplementary Material 13.

Supplementary Material 14.

## Data Availability

Immunopeptidome data are deposited to the ProteomeXchange Consortium via the PRIDE partner repository with the dataset identifier PXD055268 and 10.6019/PXD055268. The authors declare that all remaining data supporting the findings of this study are available in the main text and supplementary materials. Any other relevant data and codes are available from the corresponding author upon reasonable request.

## Abbreviations

ICI: immune checkpoint inhibition
NB: neuroblastoma
EFS: Event-Free Survival
Ab: Antibody
TMB: Tumor mutational burden
TIL: Tumor-infiltrating lymphocytes
MHC: Major histocompatibility complex
HDACi: Histone deacetylase inhibitor
APP: Antigen processing and presentation
ERAP1: Endoplasmic reticulum aminopeptidase 1
NK: Natural killer
TME: Tumor microenvironment
GFP: Green fluorescent protein
FACS: Fluorescent Activated Cell Sorting
KO: Knockout
BCA: Bicinchoninic acid
DAPI: 6-diamidino-2-phenylindole hydrochloride
ULA: Ultra-low attachment
RT: Room temperature
PI: Propidium iodide
IHC: Immunohistochemistry
IF: Immunofluorescence
sgRNAs: Single guide RNAs
pMHC: Peptide-MHC
ACN: Acetonitrile
FA: Formic acid
iRT: Indexed retention time
LC: Liquid chromatography
FDR: False discovery rate
TAPBP: Tapasin
b2m: Beta-2 microglobulin
TCR: T-cell receptor
ADR: Adrenergic
MES: Mesenchymal
TEIPP: T-cell epitopes associated with altered peptide processing
TAP: Transporter associated with antigen processing
