# Supplementary material for: Combining ERAP1 silencing and entinostat therapy to overcome resistance to cancer immunotherapy in neuroblastoma
Source: J Exp Clin Cancer Res. 2024 Oct 22;43:292. doi: 10.1186/s13046-024-03180-y (PMC11494811; doi:10.1186/s13046-024-03180-y)
Supplement: Supplementary file 1 — Supplementary Material 1. [file 13046_2024_3180_MOESM1_ESM.pdf]

Supplementary Figure 1

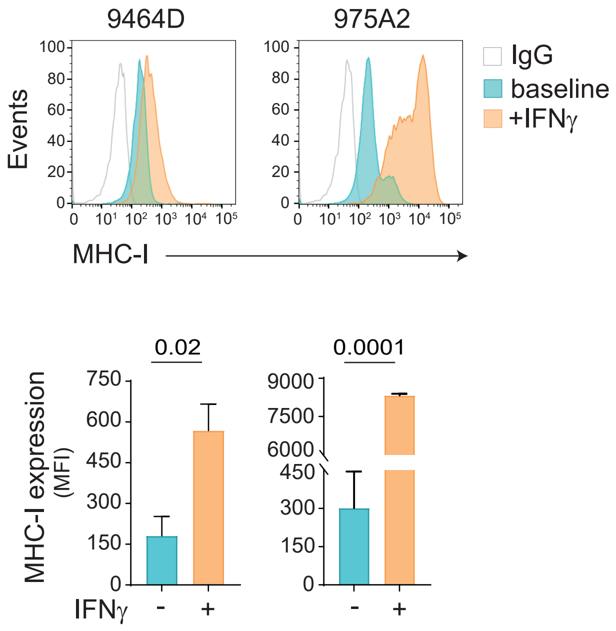

Supplementary Figure 1 related to Figure 1

Phenotype analysis of transplantable neuroblastoma 9464D and 975A2 mouse models

Representative flow-cytometry histograms of MHC class I cell surface expression in IFNγ-treated (+IFN γ) and untreated (baseline) 9464D and 975A2 tumor cells. Isotype-matched negative control antibody (IgG) is shown as grey line. Bars represent the MFI of MHC class I cell surface expression. Levels of significance for comparison between samples were determined by two-tailed Student's t test. Statistically significant P values are shown.
