# Supplementary material for: Combining ERAP1 silencing and entinostat therapy to overcome resistance to cancer immunotherapy in neuroblastoma
Source: J Exp Clin Cancer Res. 2024 Oct 22;43:292. doi: 10.1186/s13046-024-03180-y (PMC11494811; doi:10.1186/s13046-024-03180-y)
Supplement: Supplementary file 2 — Supplementary Material 2. [file 13046_2024_3180_MOESM2_ESM.pdf]

Supplementary Figure 2

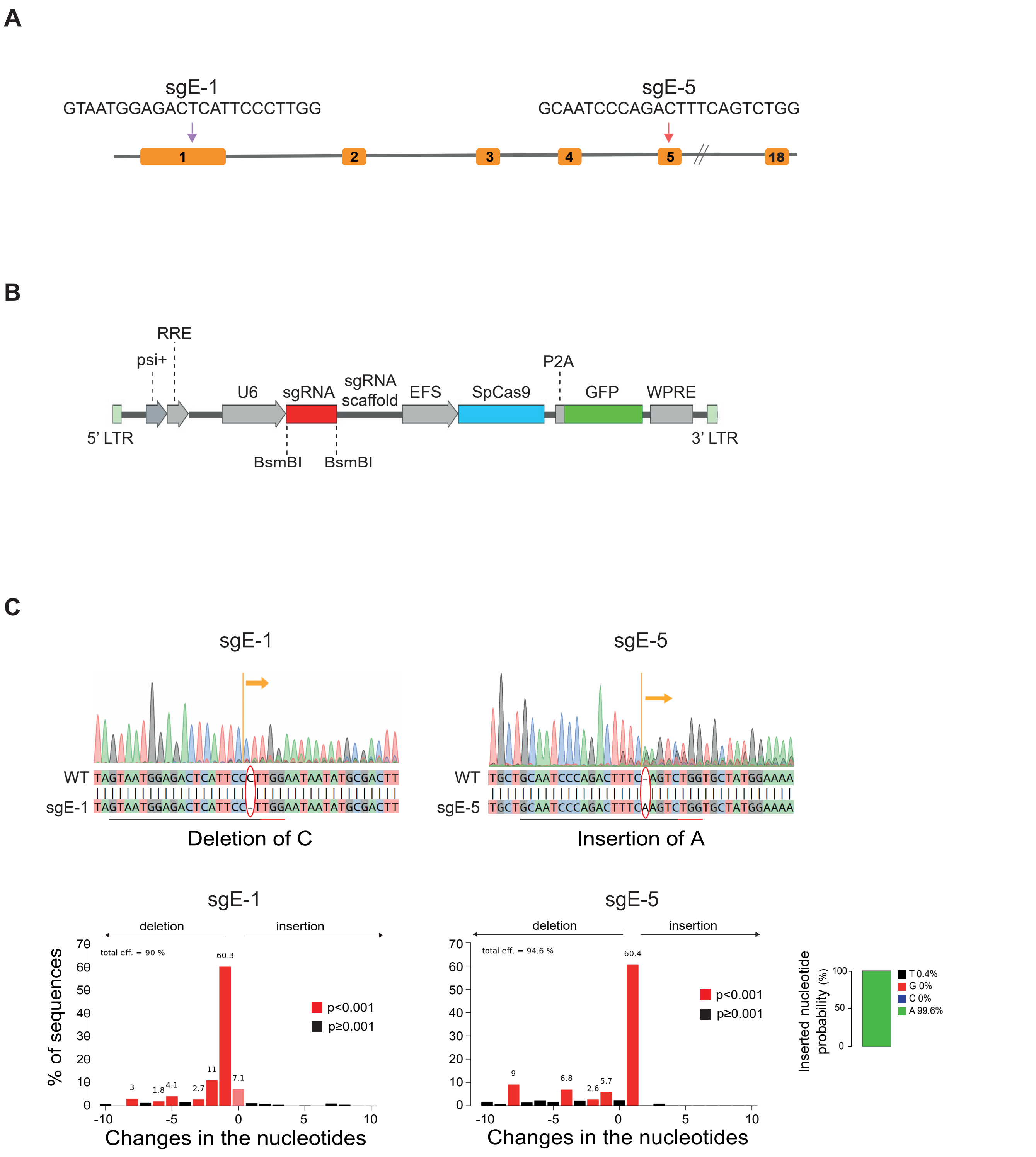

Supplementary Figure 2 related to Figure 1

ERAP1 gene editing strategy and validation of CRISPR/Cas9-mediated ERAP1 KO in 9464D cells

A Schematic representation of target site localization of the sgRNAs sgE-1 and sgE-5 on the *ERAP1* gene. Exons are illustrated as orange boxes separated by lines that represent introns. **B** Map of the Lenti-CRISPRv2GFP vector used to deliver Cas9 and sgRNAs. The red rectangle flanked by BsmBI sites is the cloning site for sgRNAs. **C** Validation of indels by bioinformatic CRISP-ID (upper panels) and TIDE (lower panels) analyses. Alignment and chromatograms from CRISP-ID tool display the size and locus of the indels. TIDE shows indels as bars indicating indel size in bp and indel frequency on the x- and y-axes, respectively.
