# Supplementary material for: Combining ERAP1 silencing and entinostat therapy to overcome resistance to cancer immunotherapy in neuroblastoma
Source: J Exp Clin Cancer Res. 2024 Oct 22;43:292. doi: 10.1186/s13046-024-03180-y (PMC11494811; doi:10.1186/s13046-024-03180-y)
Supplement: Supplementary file 3 — Supplementary Material 3. [file 13046_2024_3180_MOESM3_ESM.pdf]

Supplementary Figure 3

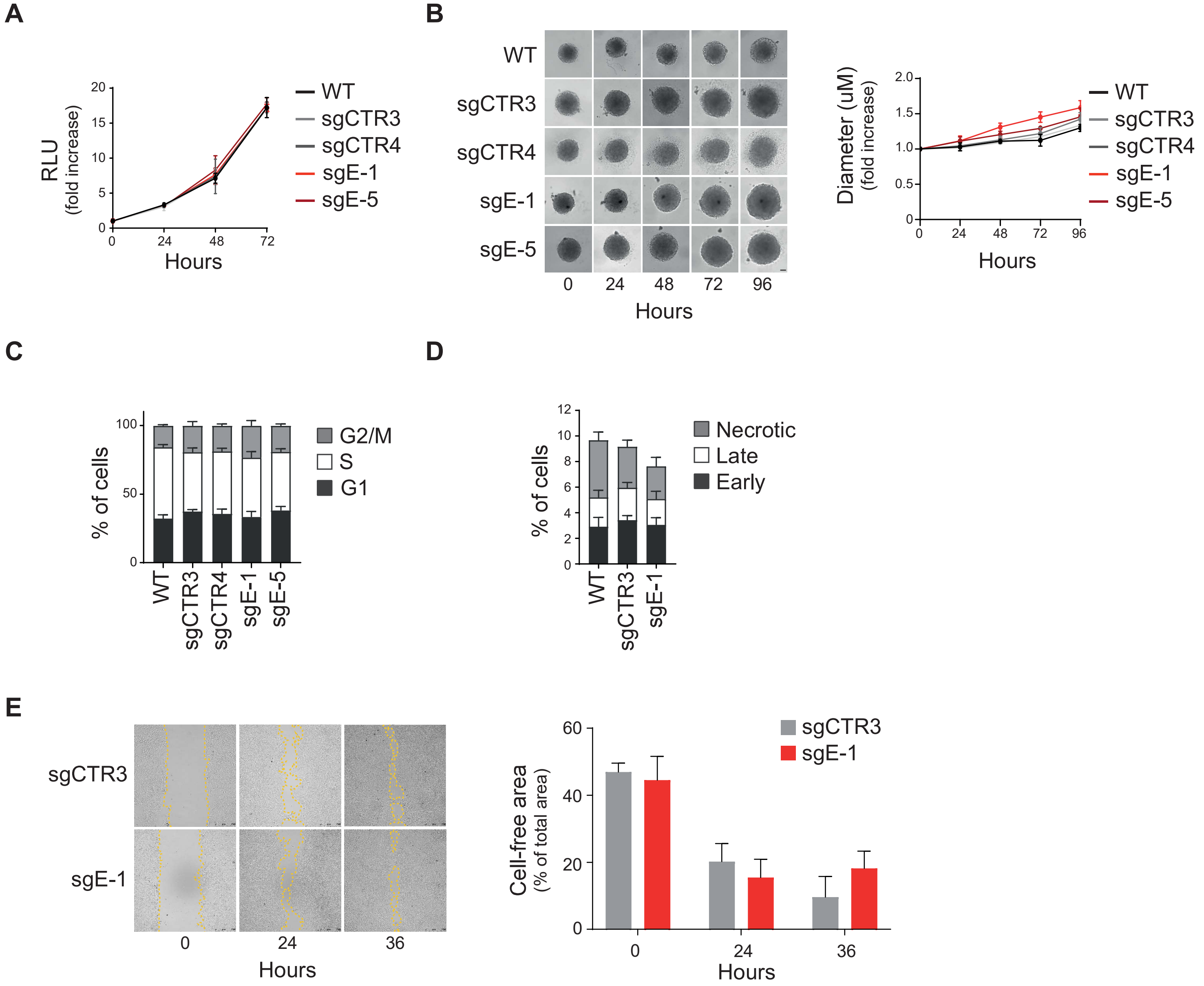

Supplementary Figure 3 related to Figure 1

Inhibition of ERAP1 does not affect the intrinsic properties of 9464D cells

A Cell proliferation of the indicated cells measured with CellTiter-Glo at different time points. Data are shown as fold over time zero of relative light units (RLU) at each time point. **B** Representative images of tumor spheroids grown on ULA plates at the indicated time points. The spheroid diameter analysis was performed with ImageJ software and shown as fold increase (right panel). **C** Cell cycle analysis of the indicated cells evaluated by flow cytometry. Histogram showing the percentage of cells in G1, S and G2/M phase. **D** Apoptotic state of the indicated cells evaluated with Annexin V/PI staining. The percentage of cells in the different phases of apoptosis are shown. **E** Representative images of cells at 0, 24 and 36 hours after wounding (left). Quantitative comparison of cell migration and wound closure in sgCTR3 and sgE-1 cells (right). Data are shown as the average value of the percentage of cell-free area.
