# Supplementary material for: Combining ERAP1 silencing and entinostat therapy to overcome resistance to cancer immunotherapy in neuroblastoma
Source: J Exp Clin Cancer Res. 2024 Oct 22;43:292. doi: 10.1186/s13046-024-03180-y (PMC11494811; doi:10.1186/s13046-024-03180-y)
Supplement: Supplementary file 4 — Supplementary Material 4. [file 13046_2024_3180_MOESM4_ESM.pdf]

Supplementary Figure 4

A

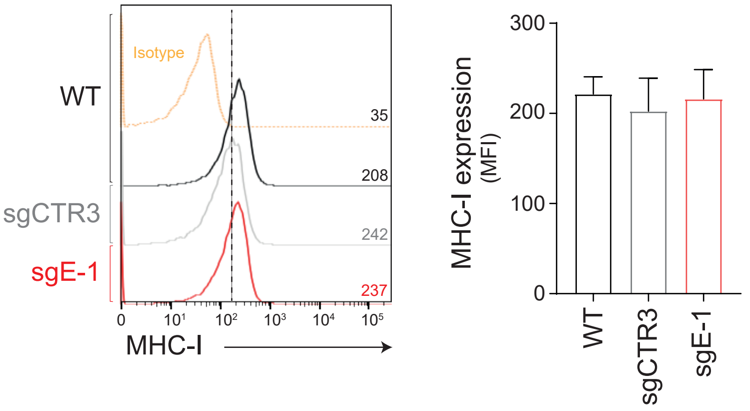

B

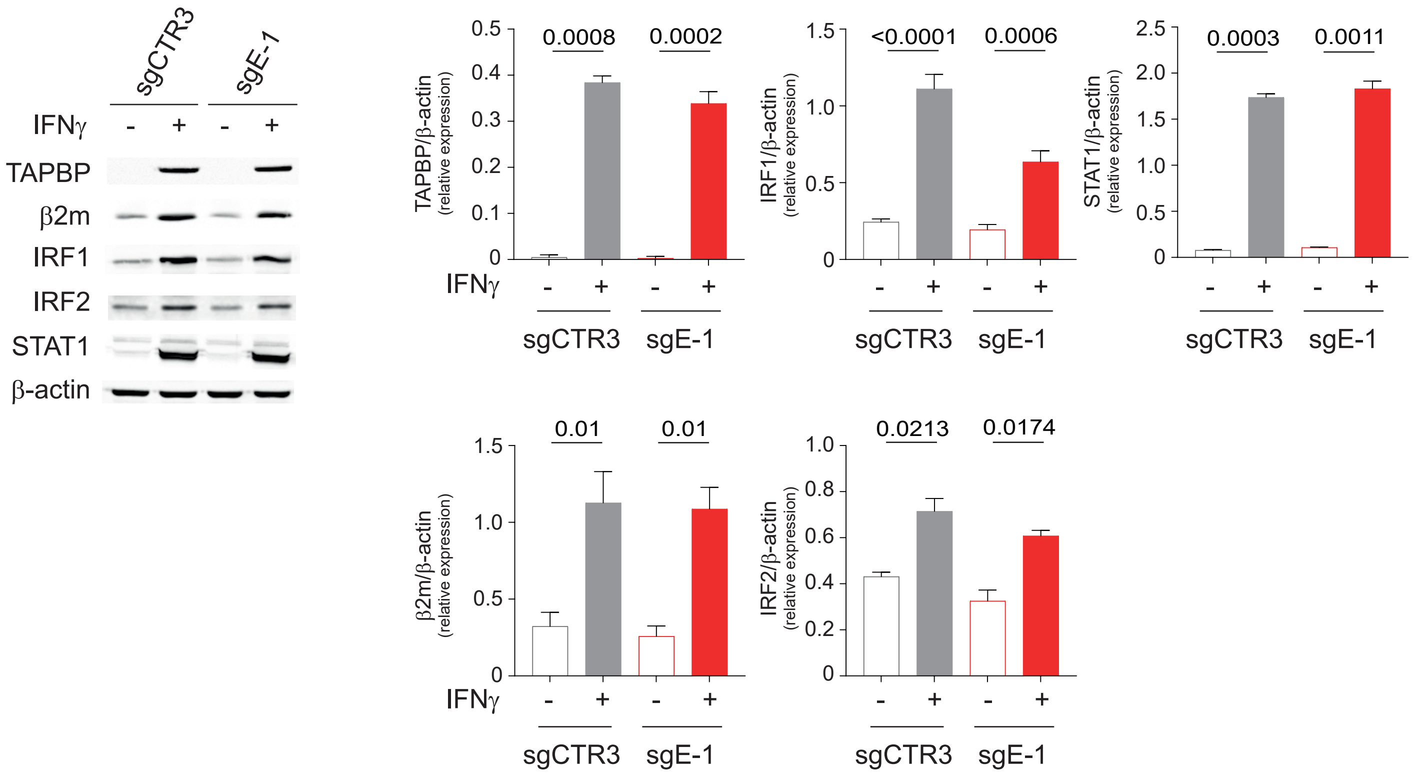

Supplementary Figure 4 related to Figure 1

Downregulation of ERAP1 does not affect IFNγ-induced expression of surface MHC class I molecules and other components of the antigen processing and IFNγ signalling pathway

A Representative flow-cytometry analysis of MHC class I cell surface expression in the indicated cells. Isotype-matched negative control antibody is shown as yellow histogram. Bars represent the MFI of MHC class I expression in the indicated cells. **B** Representative immunoblotting analysis of the expression of the components of the antigen processing and signalling pathway of IFNγ indicated in the sgCTR3 and sgE-1 cells untreated or treated with IFNγ. Densitometric analysis of β-actin-normalized ERAP1 expression from three independent experiments is shown. Levels of significance for comparison between samples were determined by ANOVA and two-tailed Student's t test. Statistically significant P values are shown.
