# Supplementary material for: Combining ERAP1 silencing and entinostat therapy to overcome resistance to cancer immunotherapy in neuroblastoma
Source: J Exp Clin Cancer Res. 2024 Oct 22;43:292. doi: 10.1186/s13046-024-03180-y (PMC11494811; doi:10.1186/s13046-024-03180-y)
Supplement: Supplementary file 5 — Supplementary Material 5. [file 13046_2024_3180_MOESM5_ESM.pdf]

Supplementary Figure 5

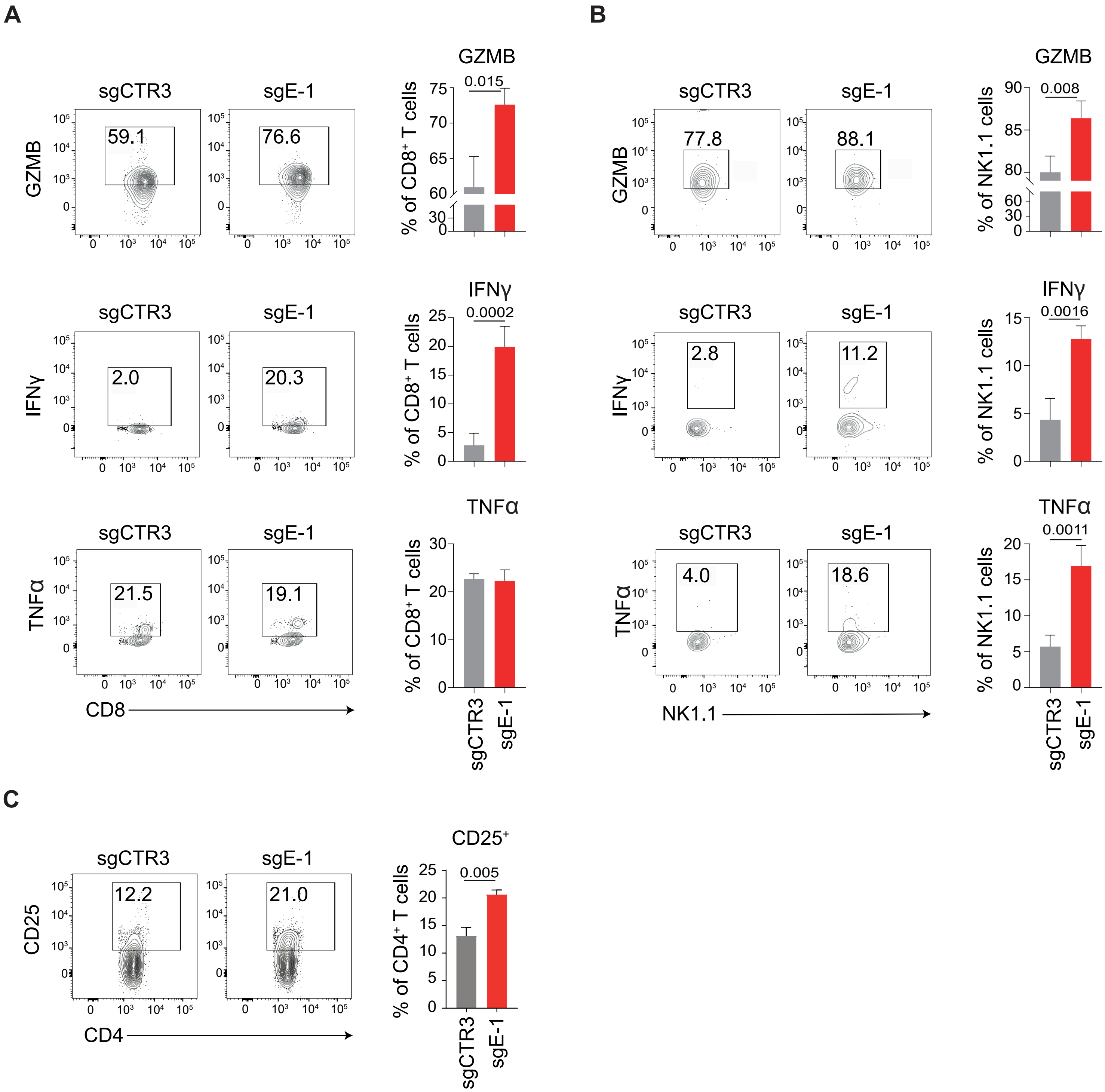

Supplementary Figure 5 related to Figure 2

Inhibition of ERAP1 in 9464D cells causes activation of CD4+ T cells, CD8+ T cells and NK cells in coculture experiments with splenocytes.

A Representative flow-cytometry analyses of CD25 expression by CD4+ T cells from splenocytes co-cultured with IFNγ-treated tumor cells for 18 hours. Bars represent the % of CD4+ T cells expressing CD25. B and C Representative flow-cytometry analyses of granzyme B (GZMB), IFNγ and TNFα expression by CD8+ T cells (B) and NK cells (C) from splenocytes co-cultured with IFNγ-treated tumor cells for 18 hours. Bars represent the % of the indicated cells expressing the specific markers. Levels of significance for comparison between samples were determined by two-tailed Student's t test. Statistically significant P values are shown..
