# Supplementary material for: Combining ERAP1 silencing and entinostat therapy to overcome resistance to cancer immunotherapy in neuroblastoma
Source: J Exp Clin Cancer Res. 2024 Oct 22;43:292. doi: 10.1186/s13046-024-03180-y (PMC11494811; doi:10.1186/s13046-024-03180-y)
Supplement: Supplementary file 6 — Supplementary Material 6. [file 13046_2024_3180_MOESM6_ESM.pdf]

Supplementary Figure 6

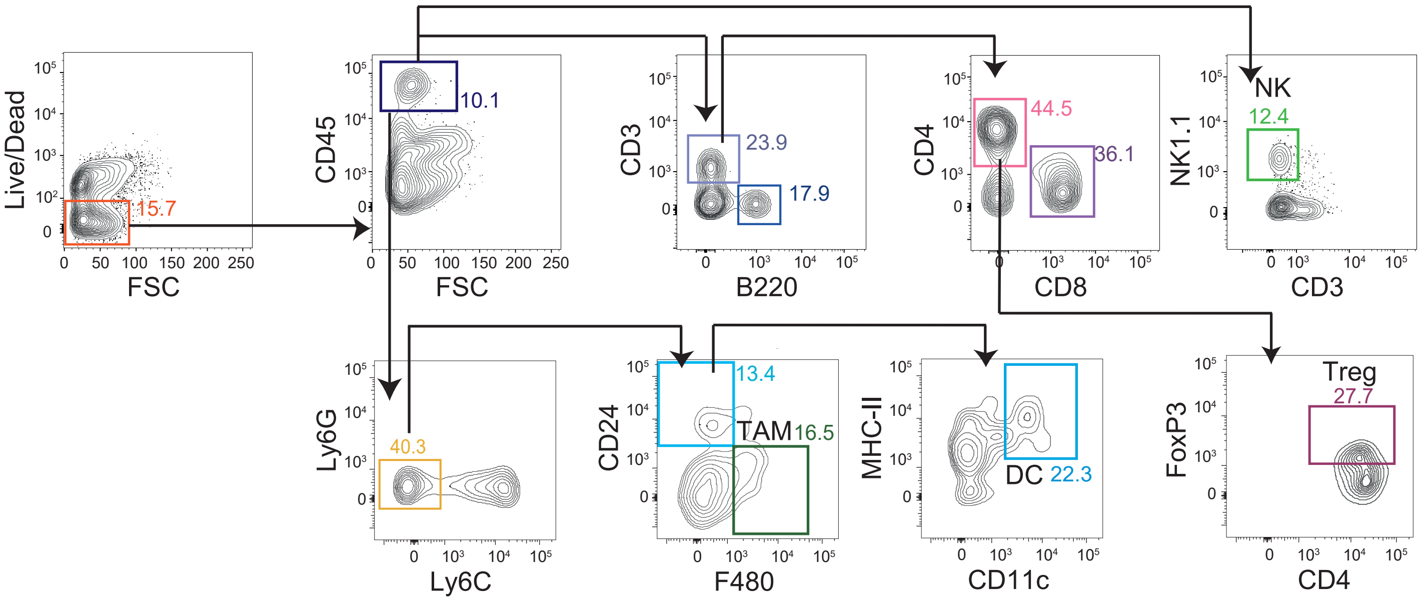

Supplementary Figure 6 related to Figure 3

Flow-cytometry analysis of tumor-infiltrating immune cells in 9464D mouse NB models.

Representative flow cytometric gating strategy for 9464D tumors to define tumor-infiltrating immune cells.
