# Supplementary material for: Combining ERAP1 silencing and entinostat therapy to overcome resistance to cancer immunotherapy in neuroblastoma
Source: J Exp Clin Cancer Res. 2024 Oct 22;43:292. doi: 10.1186/s13046-024-03180-y (PMC11494811; doi:10.1186/s13046-024-03180-y)
Supplement: Supplementary file 7 — Supplementary Material 7. [file 13046_2024_3180_MOESM7_ESM.pdf]

Supplementary Figure 7

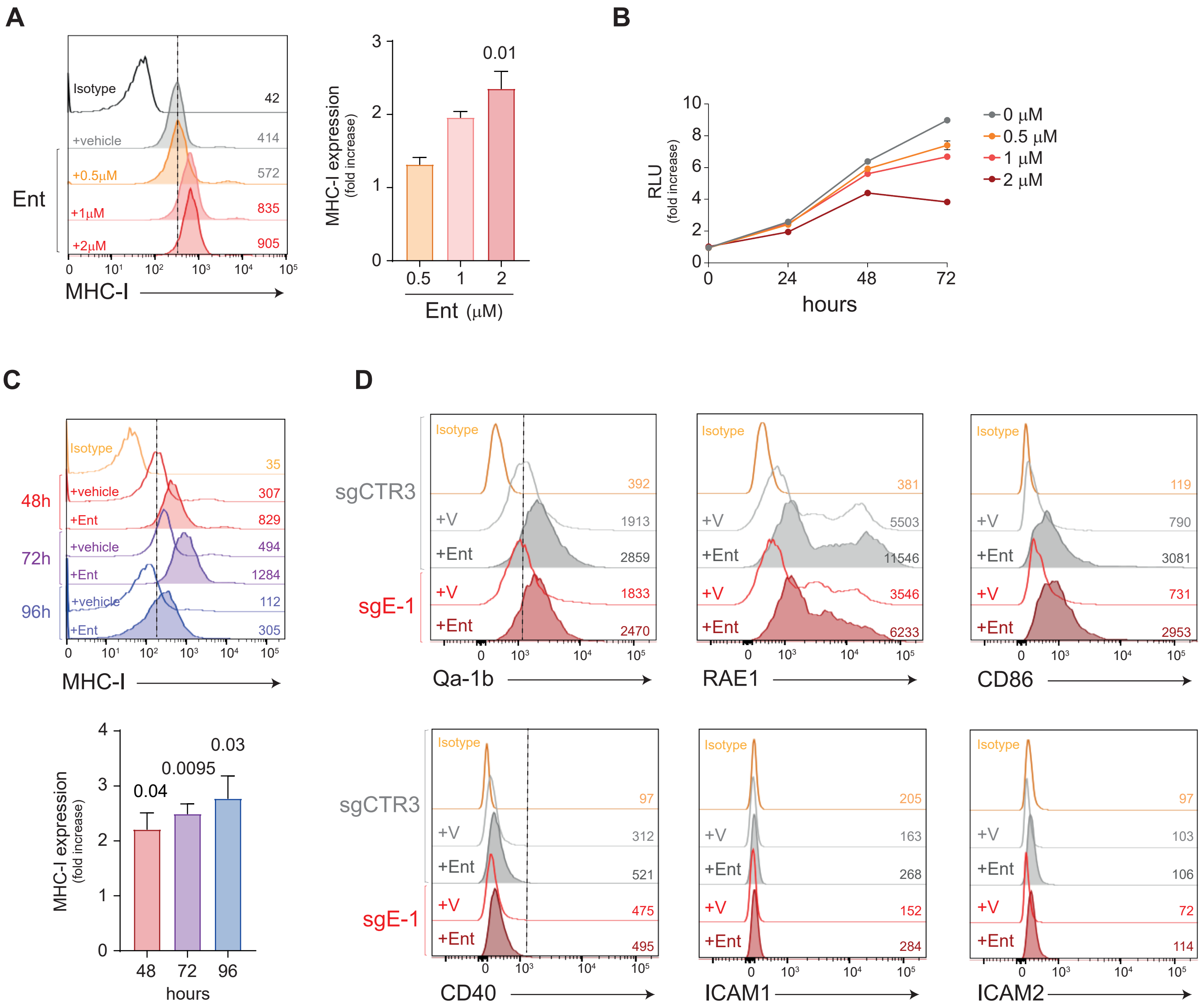

Supplementary Figure 7 related to Figure 4

Inhibition of ERAP1 affects the surface expression of classical and nonclassical MHC class I molecules of 9464D cells

A Representative flow-cytometry histograms of MHC class I expression in 9464D cells treated with entinostat at the indicated concentrations. Isotype-matched negative control antibody is shown as black histogram. Bars represent the increase in MFI of MHC class I expression in entinostat- (Ent) treated compared to untreated tumor cells. **B** Cell proliferation of 9464D cells treated with entinostat at the indicated concentration was measured with CellTiter-Glo at different time points. Data are shown as fold over time zero of relative light units (RLU) at each time point. **C** Representative flow-cytometry histograms of MHC class I expression in 9464D cells treated with entinostat at the indicated time points. Isotype-matched negative control antibody is shown as yellow histogram. Bars represent the increase in MFI of MHC class I expression in entinostat-(Ent) stimulated compared to unstimulated tumor cells. **D** Representative flow-cytometry histograms of Qa-1b, RAE1 and CD86 expression in the indicated cell lines. Isotype-matched negative control antibody is shown as yellow histogram.
