# Supplementary material for: Combining ERAP1 silencing and entinostat therapy to overcome resistance to cancer immunotherapy in neuroblastoma
Source: J Exp Clin Cancer Res. 2024 Oct 22;43:292. doi: 10.1186/s13046-024-03180-y (PMC11494811; doi:10.1186/s13046-024-03180-y)
Supplement: Supplementary file 8 — Supplementary Material 8. [file 13046_2024_3180_MOESM8_ESM.pdf]

Supplementary Figure 8

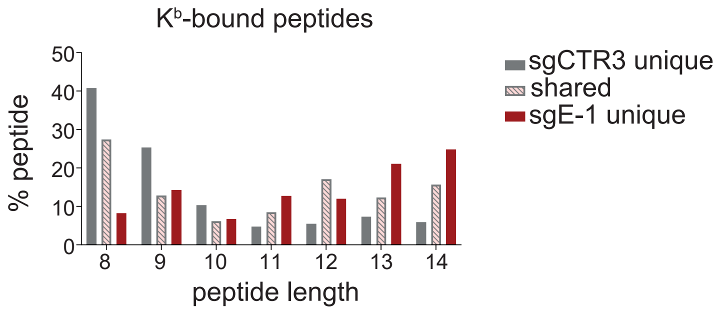

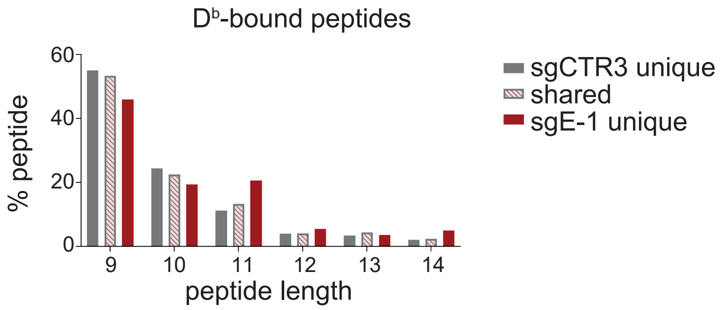

Supplementary Figure 8 related to Figure 4

Inhibition of ERAP1 leads to presentation of longer peptides by MHC class I molecules.

Unique and shared H-2K^b- and H-2D^b-bound peptides from sgCTR3 and sgE-1 cells are plotted according to their amino acid length.
