# Supplementary material for: Combining ERAP1 silencing and entinostat therapy to overcome resistance to cancer immunotherapy in neuroblastoma
Source: J Exp Clin Cancer Res. 2024 Oct 22;43:292. doi: 10.1186/s13046-024-03180-y (PMC11494811; doi:10.1186/s13046-024-03180-y)
Supplement: Supplementary file 9 — Supplementary Material 9. [file 13046_2024_3180_MOESM9_ESM.pdf]

Supplementary Figure 9

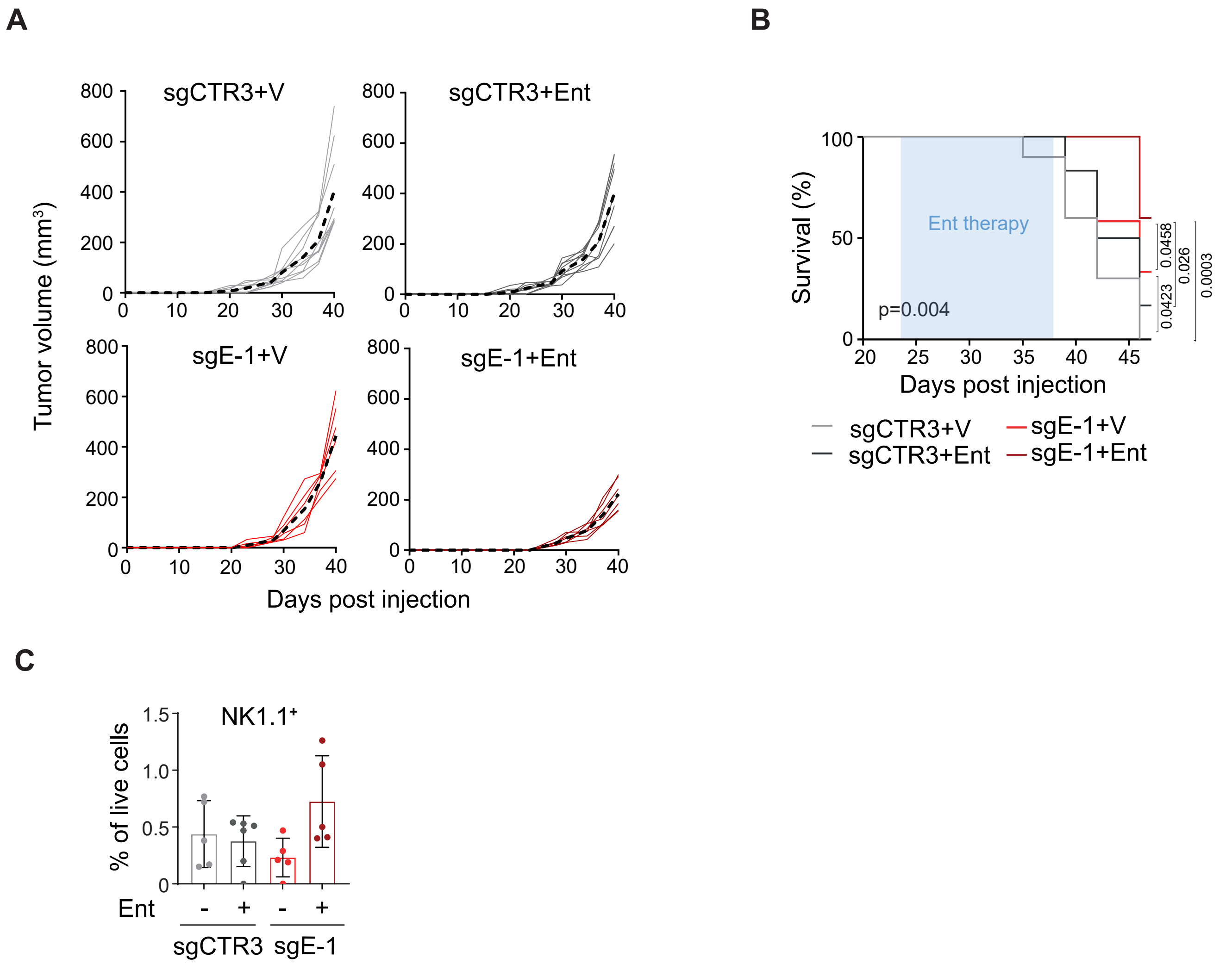

Supplementary Figure 9 related to Figure 5

Inhibition of ERAP1 in combination with entinostat treatment delays the growth of 9464D tumors and reshapes the intratumoral immune infiltrate

A Tumor growth of sgCTR3 and sgE-1 cells injected subcutaneously in C57BL/6 mice and treated as indicated. The average growth for each group is indicated with a black dotted line. **B** Survival analysis of the indicated experimental groups. **C** Flow-cytometry analysis of the NK cell content in explanted tumors at day 10 after the start of treatment ($n \geq 5$ for each group). Levels of significance for comparison between samples were determined by ANOVA and Log-rank test. Statistically significant P values are shown.
