# Supplementary material for: Combining ERAP1 silencing and entinostat therapy to overcome resistance to cancer immunotherapy in neuroblastoma
Source: J Exp Clin Cancer Res. 2024 Oct 22;43:292. doi: 10.1186/s13046-024-03180-y (PMC11494811; doi:10.1186/s13046-024-03180-y)
Supplement: Supplementary file 10 — Supplementary Material 10. [file 13046_2024_3180_MOESM10_ESM.pdf]

Supplementary Figure 10

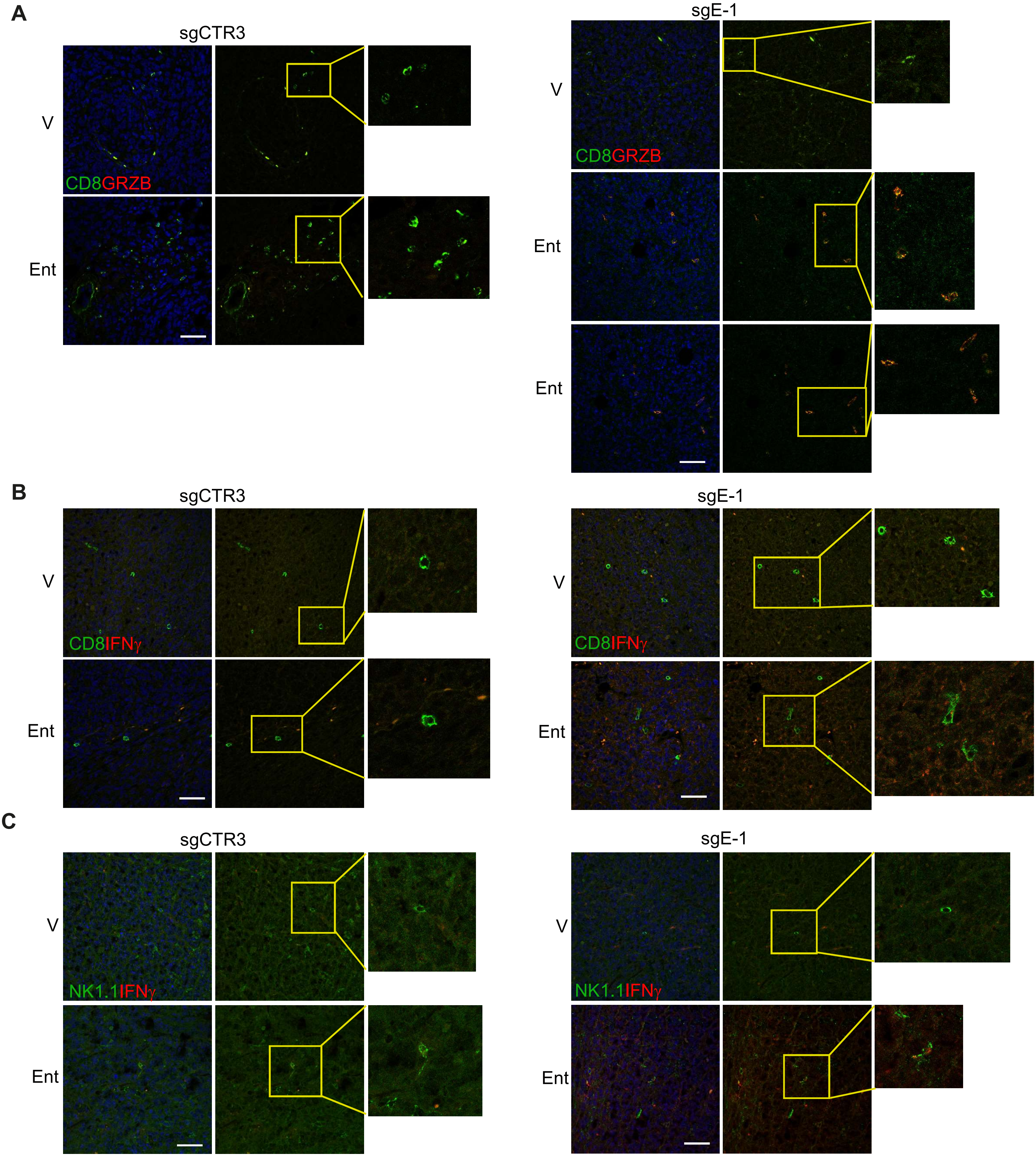


**Supplementary Figure 10 related to Figure 5**

**Inhibition of ERAP1 in combination with entinostat treatment delays the growth of 9464D tumors and reshapes the intratumoral immune infiltrate**

**A-C** Representative multiple immunofluorescence staining of the tumors analysed in Figure 5 for $CD8^+$ T cells (green) expressing (**A**) granzyme B (red) or (**B**) IFNγ (red) and NK1.1 cells (**C**) expressing IFNγ are shown at magnification 40×, scale bar 50 µm. Images with nuclei (Hoechst) are shown on the left of each panel. The yellow rectangles are higly magnified on the right-hand panels. V, vehicle control; Ent, entinostat.
