# Supplementary material for: Combining ERAP1 silencing and entinostat therapy to overcome resistance to cancer immunotherapy in neuroblastoma
Source: J Exp Clin Cancer Res. 2024 Oct 22;43:292. doi: 10.1186/s13046-024-03180-y (PMC11494811; doi:10.1186/s13046-024-03180-y)
Supplement: Supplementary file 11 — Supplementary Material 11. [file 13046_2024_3180_MOESM11_ESM.pdf]

Supplementary Figure 11

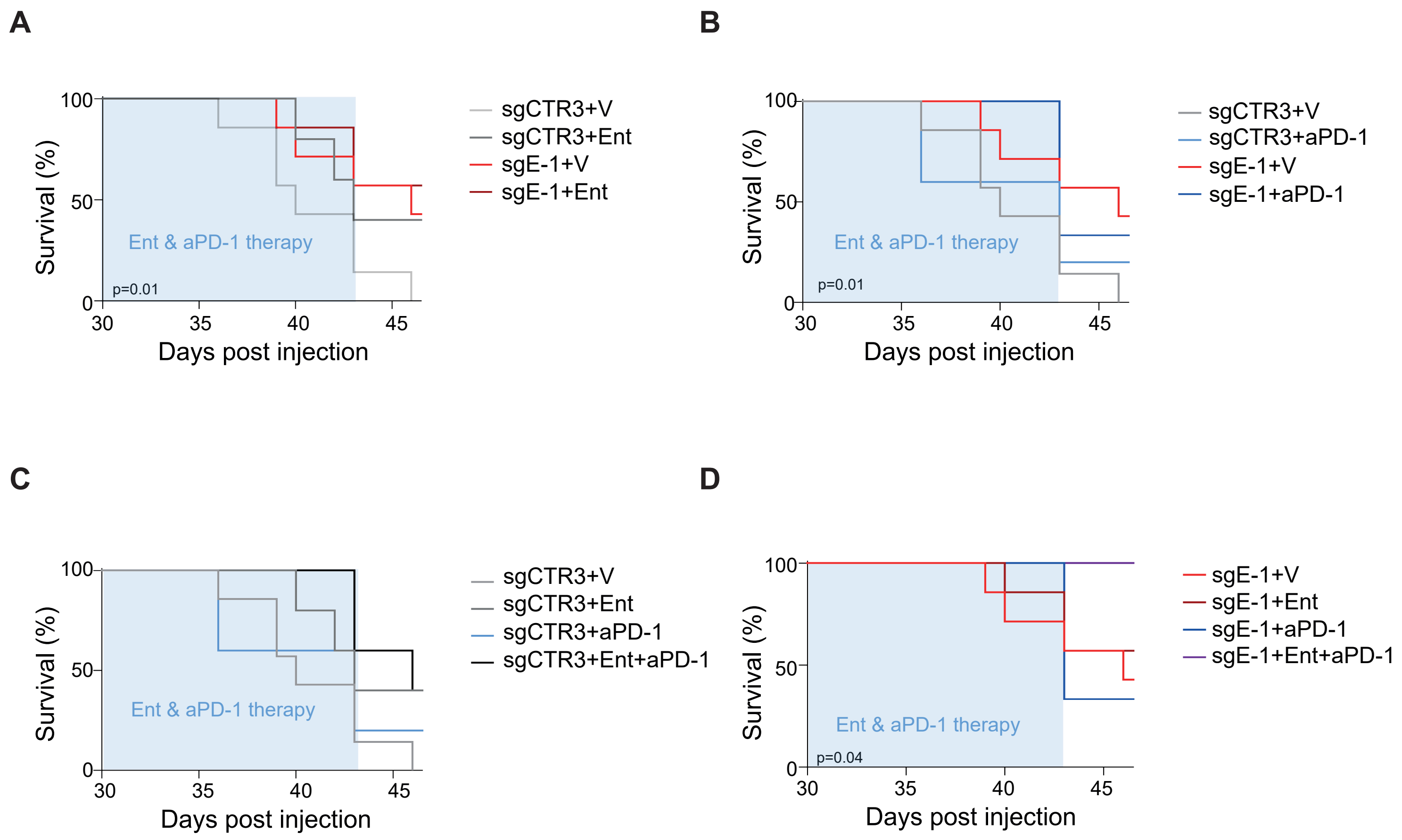

Supplementary Figure 11 related to Figure 6

ERAP1 inhibition in combination with entinostat and PD-1 blockade delays the growth of 9464D tumors

A-D Survival analysis of the indicated experimental groups. Levels of significance for comparison between samples were determined by Log-rank test. V, vehicle control; Ent, entinostat; aPD-1, anti-PD-1 antibody. Statistically significant P values are shown.
