# Supplementary material for: Combining ERAP1 silencing and entinostat therapy to overcome resistance to cancer immunotherapy in neuroblastoma
Source: J Exp Clin Cancer Res. 2024 Oct 22;43:292. doi: 10.1186/s13046-024-03180-y (PMC11494811; doi:10.1186/s13046-024-03180-y)
Supplement: Supplementary file 12 — Supplementary Material 12. [file 13046_2024_3180_MOESM12_ESM.pdf]

Supplementary Table 1. Sequence of sgRNAs used

sgRNA	sequence	Tool/reference
ERAAP sgRNA-1	GTAATGGAGACTCATTCCCT	E-CRISP
ERAAP sgRNA-5	GCAATCCCAGACTTTCAGTC	[53]
control sgRNA3	ATGTTGCAGTTCGGCTCGAT	[53]
control sgRNA4	ACGTGTAAGGCGAACGCCTT	E-CRISP

Supplementary Table 2. Oligo sequences used for PCR and Sanger sequencing

**sgRNA**	**Sequence** (5’ - 3’)
Exon 1 forward primer	TGATGATGCGTTTTGACATGCT
Exon 1 reverse primer	GTGGGCACACTTGAAGCTGAA
Exon 5 forward primer	TGAGTTGTACTCTTTCCTACCCA
Exon 5 reverse primer	TGGGAACTGGCAGACTACCT

Supplementary Table 3. List of antibodies

Marker	Coniugate	Clone	Company	Catalog	Diluition	Application
anti-mouse CD45	BV605	30-F11	BD Biosciences	563053	1:40	FACS
anti-mouse CD45	V500	30-F11	BD Biosciences	561487	1:40	FACS
anti-mouse CD3	BUV395	145-2C11	BD Biosciences	565992	1:40	FACS
anti-mouse CD4	PE CY7	GK1.5	BD Biosciences	563933	1:40	FACS
anti-mouse CD8	BUV805	53-6.7	BD Biosciences	612898	1:40	FACS
anti-mouse CD25	PE	PC61	BD Biosciences	553866	1:40	FACS
anti-mouse NK1.1	BV421	PK136	BD Biosciences	562921	1:40	FACS
anti-mouse B220	APC	RA3 6B2	BD Biosciences	553092	1:40	FACS
anti-mouse Ly6C	APC Cy7	HK1.4	BioLegend	128026	1:40	FACS
anti-mouse Ly6G	PE Cy5	IA8	BioLegend	127618	1:40	FACS
anti-mouse CD24	BV786	M1/69	BD Biosciences	744470	1:40	FACS
anti-mouse F4/80	PE	BM8	BioLegend	123109	1:40	FACS
anti-mouse MHC-II	AF700	M5/114.15.2	BioLegend	107614	1:40	FACS
anti-mouse CD11c	BUV395	N418	BioLegend	744180	1:40	FACS
anti-mouse CD69	APC-Cy7	H1.2F3	BD Biosciences	561240	1:40	FACS
FVS620*	FSV620	/	BD Horizon	564996	1:40	FACS
anti-mouse $H2K^b/H2\text{-}D^b$	PE	28-8-6	BioLegend	114607	1:40	FACS
anti-mouse Qa-1b	PE	6A8.6F10.1 A6	BD Biosciences	566640	1:40	FACS
anti-mouse CD86	PE-Cy7	B7-2	eBioscience	25-0862-82	1:40	FACS
anti-mouse CD40	PE	1C10	eBioscience	12-0401-82	1:40	FACS
anti-mouse RAE-1 Pan Specific	PE	REA723	Miltenyi	130-111-469	1:40	FACS
anti-mouse ICAM1	FITC	REA171	Miltenyi	130-132-192	1:40	FACS
anti-mouse ICAM2	APC	REA745	Miltenyi	130-112-030	1:40	FACS
anti-IFNγ	PE-Cy7	XMG1.2	BD Biosciences	561040	1:40	FACS
anti-TNFα	APC-Cy7	MP6-XT22	BD Biosciences	560658	1:40	FACS
anti-Granzyme B	APC	GB11	eBiosciences	17-8898-82	1:40	FACS
Rabbit anti-SOX9	/	D8G8H	Cell Signaling	82630	1:200	IHC
Rabbit anti-CD8	/	D4W2Z	Cell Signaling	98941	1:100	IHC/IF
Rat anti-CD8	/	53-6.7	ThermoFisher Scientific	MA1-10301	1:50	IF
Mouse anti-NK1.1	/	PK136	ThermoFisher	MA1-70100	1:100	IF
Rabbit anti-GranzymeB	/	D2H2F	Cell Signaling	17215	1:100	IF

Rabbit anti-IFNγ	CF594	/	Biorbyt	Orb10878-CF594	1:50	IF
Goat anti-rabbit IgG	Alexa Fluor™ 594	/	ThermoFisher Scientific	A-11037	1:500	IF
Goat anti-mouse IgG	Alexa Fluor™ 488	/	ThermoFisher Scientific	A-11017	1:500	IF
Donkey anti-rat IgG	Alexa Fluor™ Plus 647	/	ThermoFisher Scientific	A48272	1:500	IF
EnVision FLEX	HRP	/	Agilent Dako	K8000	/	IHC
Mouse anti-ERAP1[#]	/	6H9	/	/	1:2000	WB
Mouse anti-β-Actin	/	C4	Santa Cruz	sc-47778	1:2000	WB
Rabbit anti-TAPBP	/	/	Proteintech	30500-1-AP	1:1500	WB
Rabbit anti-Beta-2-Microglobulin	/	/	Proteintech	13511-1-AP	1:4000	WB
Rabbit anti-IRF1	/	/	Proteintech	11335-1-AP	1:1000	WB
Rabbit anti-IRF2	/	/	Proteintech	12525-1-AP	1:2000	WB
Rabbit anti-STAT1	/	/	Proteintech	10144-2-AP	1:5000	WB
Goat anti-mouse IgG	(H+L)-HRP	/	Bio-Rad	1706516	1:3000	WB
Goat anti-rabbit IgG	(H+L)-HRP	/	Bio-Rad	1706515	1:10000	WB
anti-mouse H-2K^b	/	Y3	ATCC	HB-176		IP
anti-mouse H-2D^b	/	28.14.8s	ATCC	HB-27		IP

*FVS: Fixable Viability Stain
[#]Hybridoma-produced antibody
