# Supplementary material for: Combining ERAP1 silencing and entinostat therapy to overcome resistance to cancer immunotherapy in neuroblastoma
Source: J Exp Clin Cancer Res. 2024 Oct 22;43:292. doi: 10.1186/s13046-024-03180-y (PMC11494811; doi:10.1186/s13046-024-03180-y)
Supplement: Supplementary file 14 — Supplementary Material 14. [file 13046_2024_3180_MOESM14_ESM.pdf]

Supplementary Table 5. Number of identified peptides of 8-14 amino acids

	$H\text{-}2K^b$			$H\text{-}2D^b$		
**Condition**	**Total**	**Binders**	**Unique**	**Total**	**Binders**	**Unique**
sgCTR3	1703	483	272	4004	2225	681
sgE-1	1713	344	133	3772	2063	519
Total	3416	827	405	7776	4288	1200
